# Supplementary material for: Transient inhibition of p53 enhances prime editing and cytosine base-editing efficiencies in human pluripotent stem cells
Source: Nat Commun. 2022 Oct 27;13:6354. doi: 10.1038/s41467-022-34045-7 (PMC9613702; doi:10.1038/s41467-022-34045-7)
Supplement: Supplementary file 1 — Supplementary Information [file 41467_2022_34045_MOESM1_ESM.pdf]

Supplementary Information

Transient inhibition of p53 enhances prime editing and cytosine base editing efficiencies in human pluripotent stem cells

Authors: Mu Li[1,#], Aaron Zhong[1,#], Youjun Wu[1,#], Mega Sidharta[1,#], Michael Beaury[1], Xiaolan Zhao[2], Lorenz Studer[3,*], Ting Zhou[1,*]

Affiliations:

[1] The SKI Stem Cell Research Facility, The Center for Stem Cell Biology and Developmental Biology Program, Sloan-Kettering Institute for Cancer Research, 1275 York Avenue, New York, NY, 10065, USA.

[2] Molecular Biology Program, Memorial Sloan Kettering Cancer Center, New York, NY 10065, USA.

[3] The Center for Stem Cell Biology and Developmental Biology Program, Sloan-Kettering Institute for Cancer Research, 1275 York Avenue, New York, NY, 10065, USA.

[#] These authors contributed equally: Mu Li, Aaron Zhong, Youjun Wu, Mega Sidharta.

* e-mail: Lorenz Studer (studerl@mskcc.org); Ting Zhou (zhout@mskcc.org).

Supplementary Fig. 1

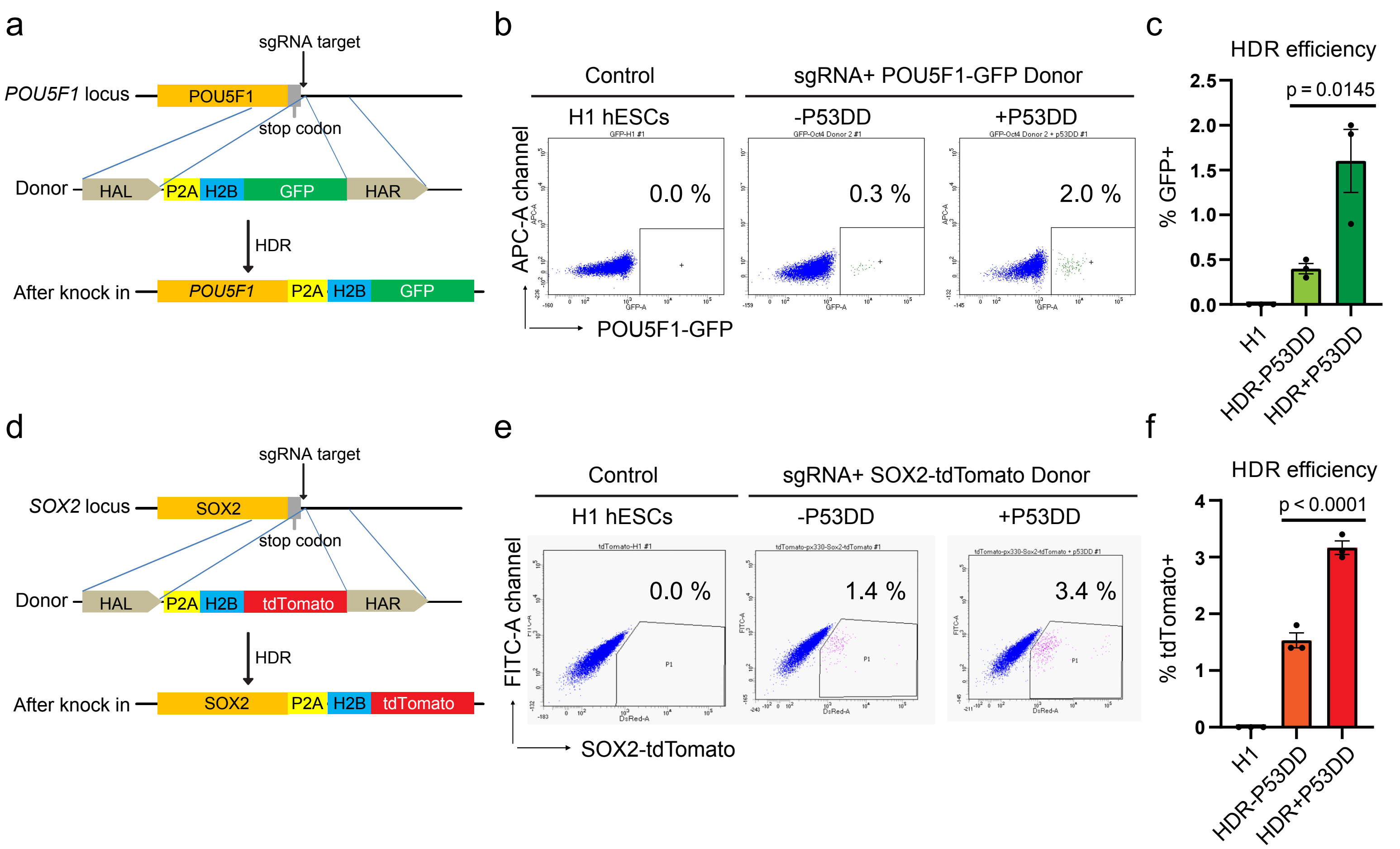

Supplementary Figure 1. p53DD increased efficiency for CRISPR/Cas9 based HDR.

a, Schematics of performing the POU5F1-P2A-H2B-GFP knock-in in H1 hESCs. POU5F1-sgRNA and POU5F1 donor constructs were electroporated into H1 hESCs for the knock-in. The POU5F1-sgRNA construct was designed targeting a region close to the stop codon of POU5F1 gene. A donor construct containing a left homology arm, followed by a P2A-H2B-GFP cassette, and a right homology arm was used as template for HDR. In this system, the GFP expression would be turned on when HDR happens in H1 hESCs.

b, c, Flow cytometry analysis (**b**), and the quantification of GFP+ % at 72 h post-electroporation with the POU5F1-sgRNA and POU5F1 donor constructs, with or without p53DD. Plain H1 hESCs were used as a negative control (**c**). n = 3 independent electroporation reactions for each condition. Values presented as mean ± S.D. p values were calculated by ordinary one-way ANOVA.

d, Schematics of performing the SOX2-P2A-H2B-tdTomato knock-in in H1 hESCs. The design is similar as POU5F1 KI shown as in (a). In this system, the tdTomato expression would be turned on when HDR happens in H1 hESCs.

e, f, Flow cytometry analysis (**e**), and the quantification of tdTomato+ % at 72 h post-electroporation with the SOX2-sgRNA and SOX2 donor construct, with or without p53DD. H1 hESCs was used as a negative control (**f**). n= 3 independent electroporation reactions for each condition. Values presented as mean ± S.D. p values were calculated by ordinary one-way ANOVA.

The source data of **c, f** are provided in Source Data file.

Supplementary Fig. 2

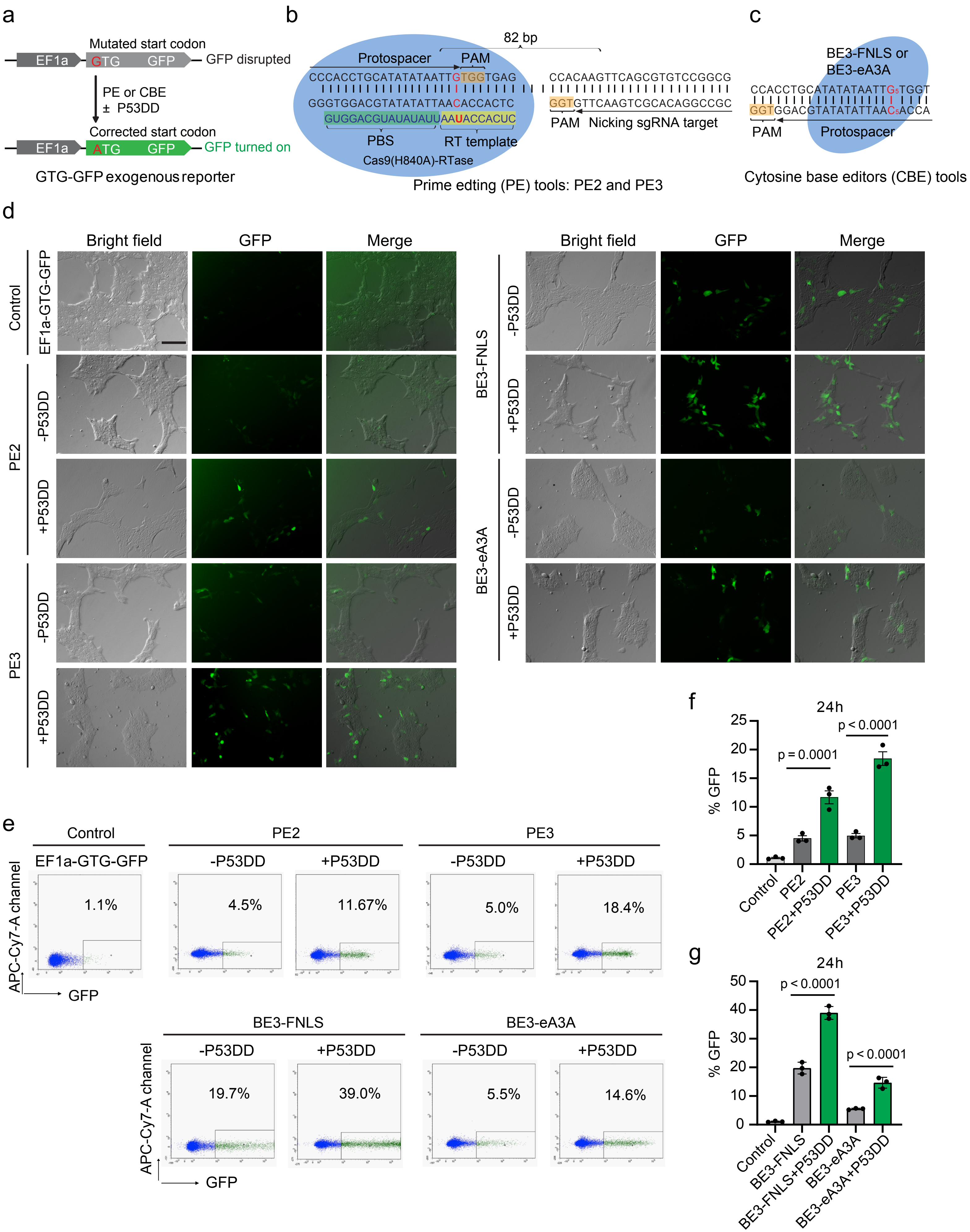

Supplementary Figure 2. p53DD increase the PE or CBE- mediated editing efficiency identified in an GTG-GFP vector reporter system.

a, Schematics of using the "GTG-GFP" exogenous reporter vector to evaluate editing efficiency of PE and CBE with or without p53DD. The vector contains a GFP cassette with a mutated start codon (GTG) under control of an EF1α promoter. PE or CBE tools was designed to covert the "GTG" to "ATG" to turn on GFP expression.

b, Schematics of pegRNA design and PE tools for editing the "GTG-GFP" vector. PegRNA is composed of a protospacer sequence, primer- binding site (PBS) and a reverse transcript (RT) template that includes the edits (G-to-A indicated here). PE2 tools includes the pegRNA and PE2 enzyme (illustrated as a blue oval). PE3 tools includes an additional nicking sgRNA targeting the complementary DNA strand at 82 bp downstream from the pegRNA nicking site.

c, Schematics of sgRNA design and CBE tools for editing the "GTG-GFP" vector. The sgRNA target for CBE was designed to edit the position 5 "C-to-T" at the non-coding strand to achieve GTG-ATG switch in the coding strand of the "GTG-GFP" vector. CBE tools include a sgRNA and a CBE enzyme (BE3-FNLS or BE3-eA3A, illustrated as a blue oval).

d. Representative bright field and fluorescent images of cells at 24 h post-electroporation with each condition. Scale bars, 250 μm. n= 3 independent electroporation reactions for each condition.

e-g, Flow cytometry analysis of GFP+ cell population at 24 h post-electroporation with each condition (**e**), and the quantification of GFP+ % in PE (**f**), and CBE (**g**) editing conditions with or without p53DD. n= 3 independent electroporation reactions for each condition. Values presented as mean ± S.D. p values were calculated by ordinary one-way ANOVA. The source data of **f, g** are provided in Source Data file.

Supplementary Fig. 3

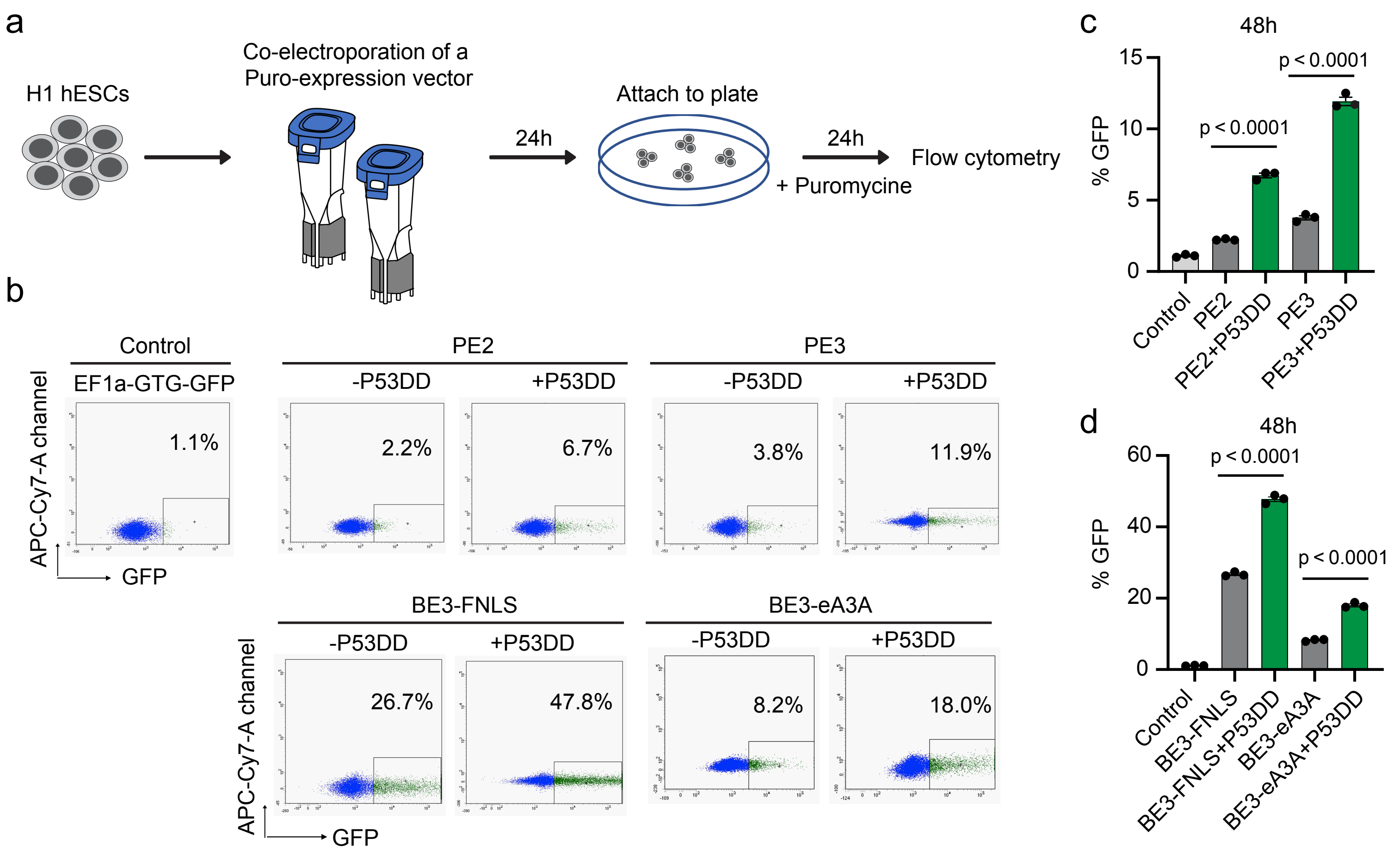

Supplementary Figure 3. p53DD increase PE or CBE- mediated editing efficiency in an conditions with minimized electroporation variation.

a, Schematics of adding an additional Puromycin-expression vector (PUC57-puro) for co-electroporation with different conditions. 0.5 µg/ml puromycin was added into the Stemflex medium for selection at 24h post-electroporation and after the cells attached to the cell culture plate. The GFP turn-on efficiency was evacuated at 48 h post-electroporation using flow cytometry.

b-d, Flow cytometry analysis of GFP+ cell population at 48 h post-electroporation with each condition (**b**), and the quantification of GFP+ % in PE (**c**), and CBE (**d**) editing conditions with or without p53DD. n= 3 independent electroporation reactions for each condition. Values presented as mean ± S.D. p values were calculated by ordinary one-way ANOVA.

The source data of **c, d** are provided in Source Data file.

Supplementary Fig. 4

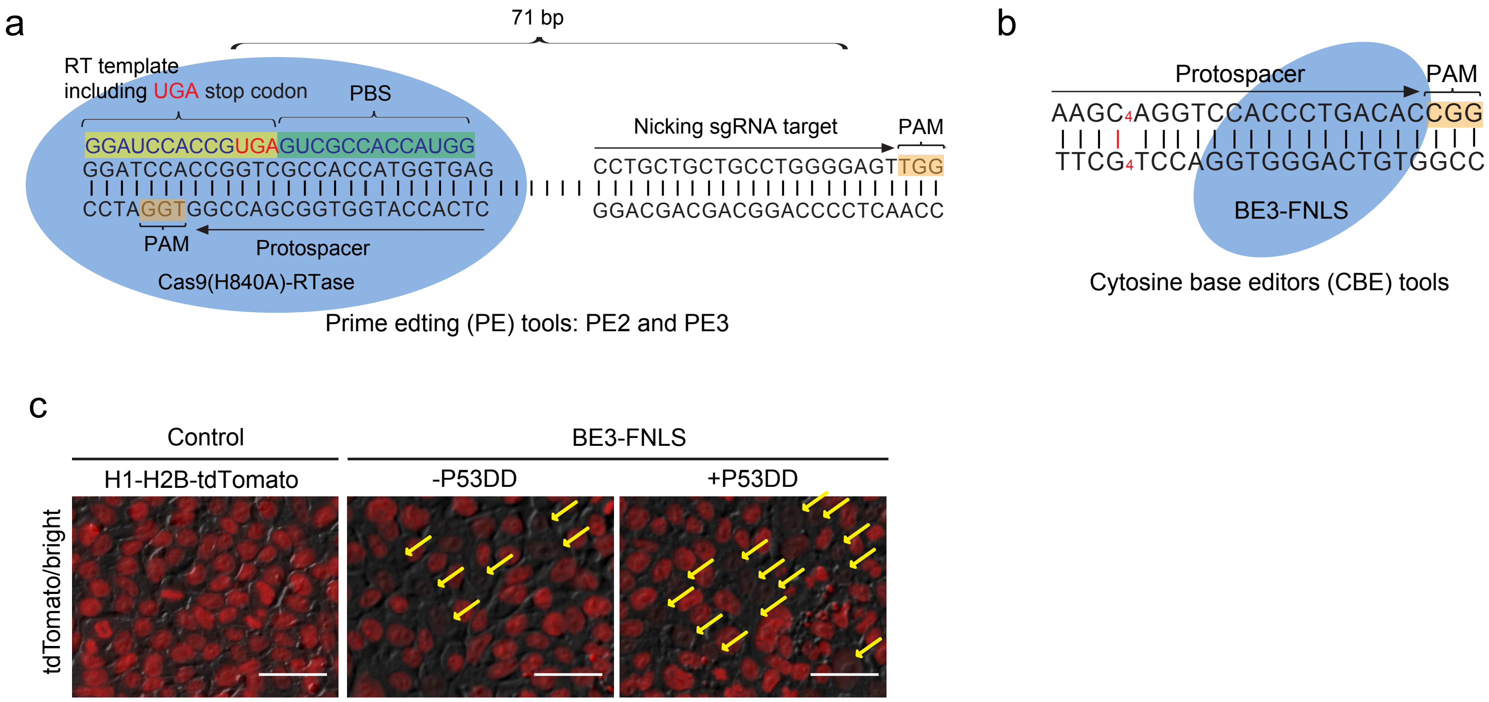

Supplementary Figure 4. A designed "H2B-tdTomato" endogenous reporter for evaluating PE and CBE genome editing efficiency.

a, Schematics of pegRNA design and PE tools for TGA insertion in the H1-SOX2-P2A-H2B-tdTomato reporter line. The PegRNA is composed of a protospacer sequence that targets to the H2B-tdTomato sequence, a primer- binding site (PBS) and a reverse transcript (RT) template that includes the edit (i.e., TGA insertion). The PE2 tools include a pegRNA and a PE enzyme (Cas9-H840A fused to a reverse transcriptase, illustrated as a blue oval here). The PE3 tools include an additional nicking sgRNA targeting the complementary DNA strand at 71 bp downstream from the pegRNA nicking site.

b, Schematics of the sgRNA design and CBE tools for C-to-T conversion in the H1-SOX2-P2A-H2B-tdTomato reporter line. The sgRNA target for CBE was designed to edit the position 4 "C-to-T" at the coding strand to generate a stop codon inside the H2B-tdTomato open reading frame. The CBE tools include the sgRNA and a CBE enzyme (BE3-FNLS) which was illustrated as a blue oval here.

c, Representative bright field and tdTomato fluorescent images (merged) of edited H1-SOX2-P2A-H2B-tdTomato reporter line using CBE, with or without p53DD. The unedited H1-SOX2-P2A-H2B-tdTomato reporter line was used as a control. Yellow arrows point to the cells that whose tdTomato expression was turned-off as a result of gene editing. Scale bars, 100 μm. n= 3 independent electroporation reactions for each condition.

Supplementary Fig. 5

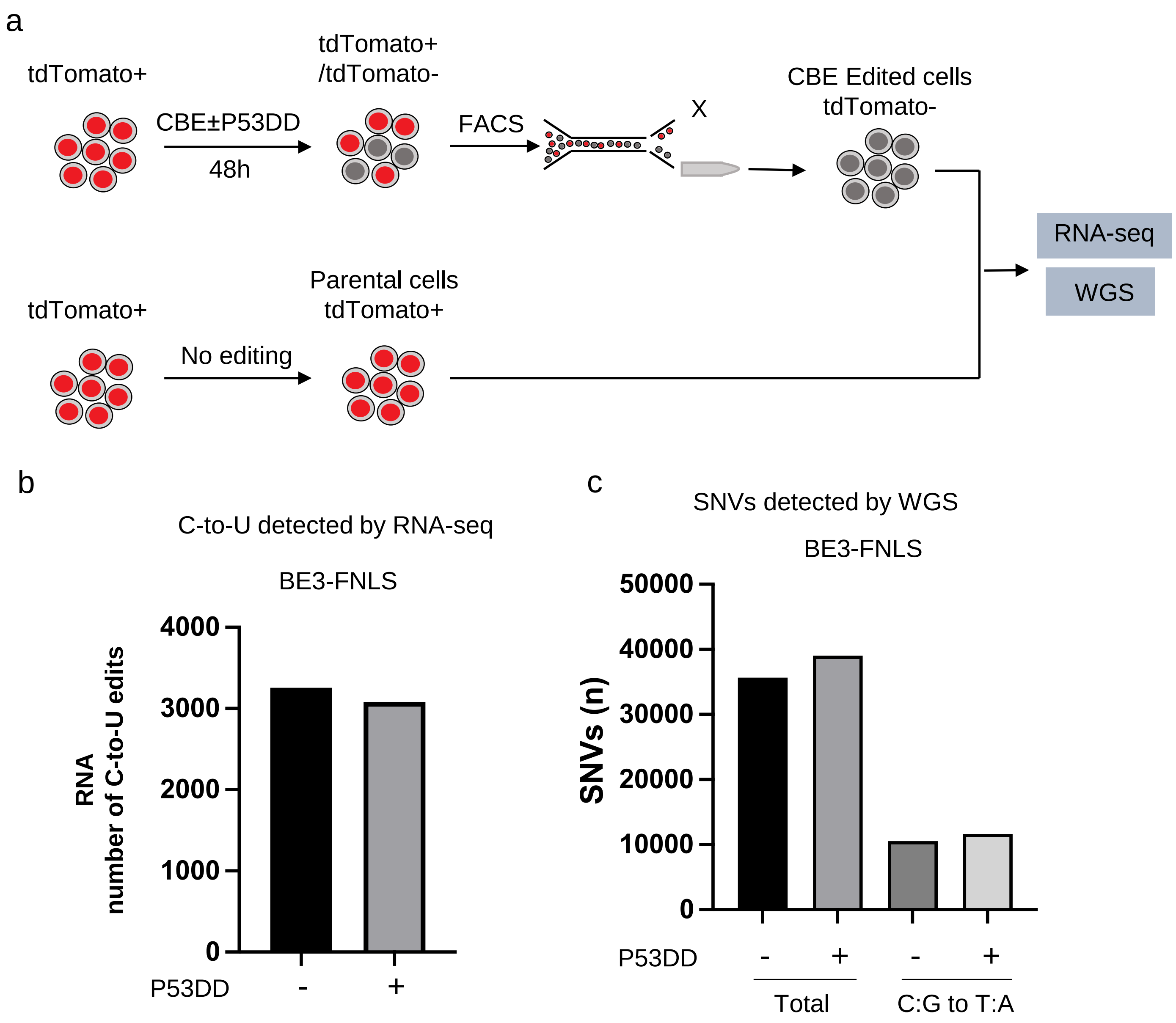

Supplementary Figure 5. Transcriptome-wide and genome-wide off-target effects were comparable in CBE with or without p53DD.

a. Schematic overview of experimental design used to identify mutations in the H1-H2B-tdTomato cells edited with CBE with or without p53DD by using transcriptome-wide and genome-wide sequencing. Edited cell population (tdTomato negative) was isolated by FACS sorting for RNA-seq and whole genome sequencing (WGS) analysis. Mutations were identified by comparing the sequence of edited cells to that of parental non-edited cells.

b. Number of C-to-U edits induced by CBE (BE3-FNLS) with or without p53DD identified in RNA-seq analysis.

c. Total number of single nucleotide variants (SNVs) and number of C:G to T:A SNVs in the CBE edited cells with or without p53DD treatment identified in WGS analysis.

The source data of **b, c** are provided in Source Data file.

Supplementary Fig. 6

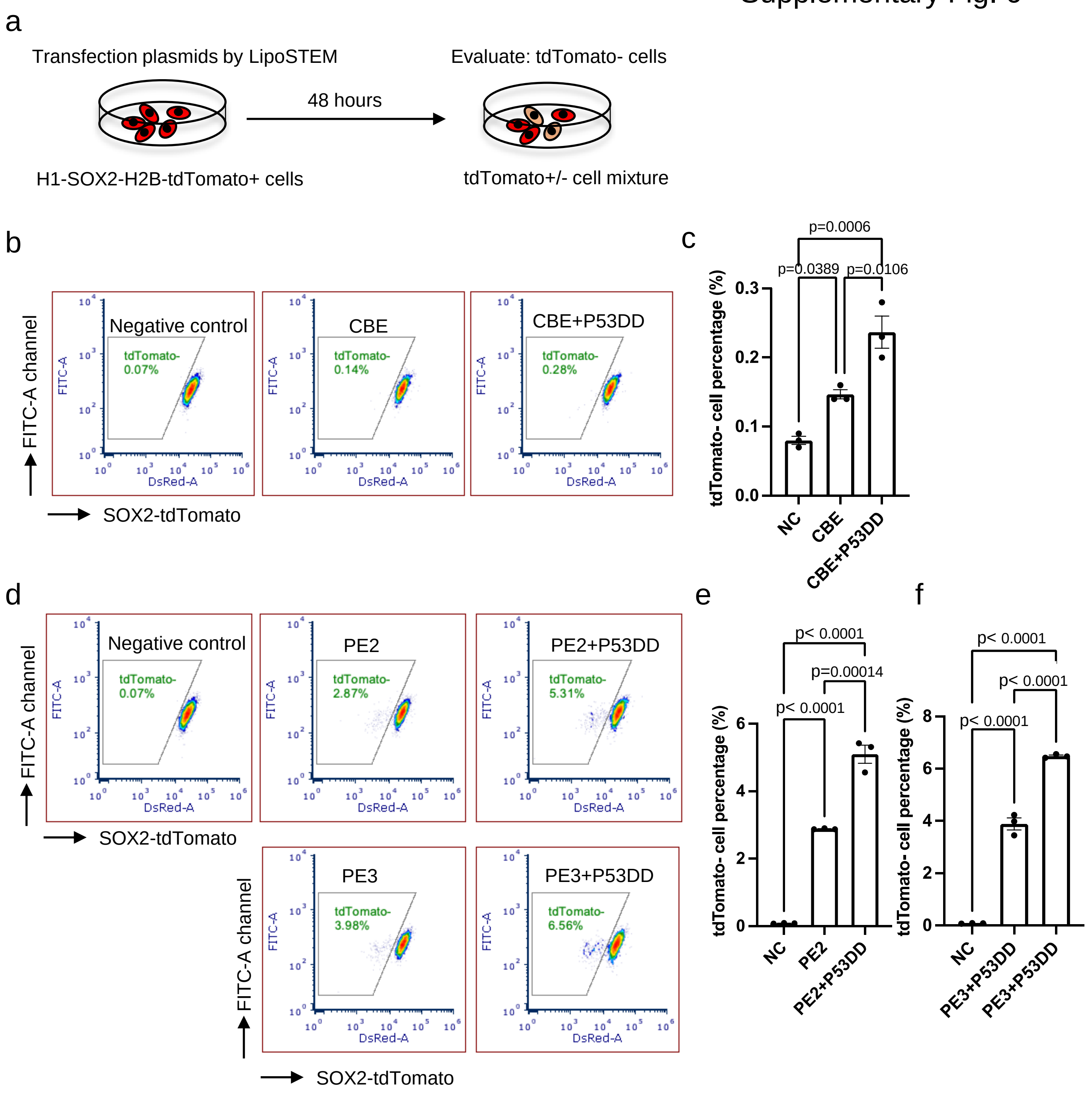

Supplementary Figure 6. p53DD increased the PE and CBE editing efficiencies via LipoStem delivery method.

a, Schematics of evaluating the effects of adding the p53DD plasmid into the PE and CBE editing tool kits respectively via LipoStem co-transfection, in “H1-H2B-tdTomato” endogenous reporter cells. The % of tdTomato negative cells was evaluated at 48h post-transfection as the read out. LipoStem transfection reagent were purchased from Thermo Fisher Scientific (STEM00008).

b, c, Flow cytometry analysis (**b**), and the quantification of tdTomato negative population % at 48 h post-electroporation with CBE tools (sgRNA and BE3-FNLS), with or without p53DD. Unedited H1-H2B-tdTomato cell line was used as a control (**c**). n= 3 independent LipoStem transfection reactions for each condition. Values presented as mean ± S.D. p values were calculated by ordinary one-way ANOVA.

d-f, Flow cytometry analysis (**d**), and quantification of the tdTomato negative population % at 48 h post-electroporation with PE2 or PE3 tools, with or without p53DD. Unedited H1-H2B-tdTomato cell line was used as a control (**e, f**). n= 3 independent LipoStem transfection reactions for each condition. Values presented as mean ± S.D. p values were calculated by ordinary one-way ANOVA.

The source data of **c, e, f** are provided in Source Data file.

Supplementary Fig. 7

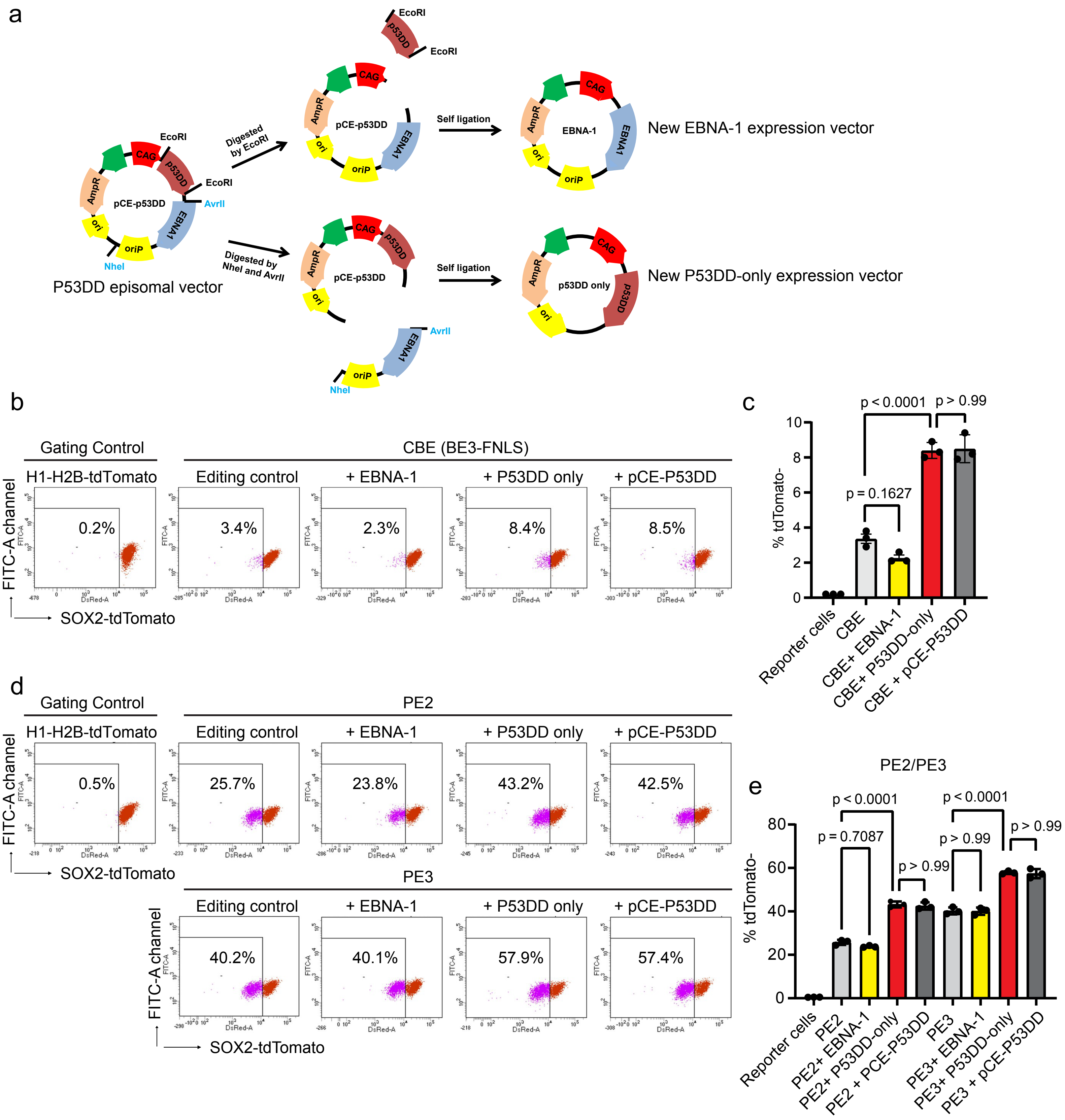

Supplementary Figure 7. p53DD-only fragment, but not the EBNA1 fragment, increased the PE and CBE editing efficiencies.

a, A scheme showing the re-construction of the original pCE-p53DD episomal vector. To generate a vector without p53DD part but only carries the EBNA-1 expression fragment, EcoR1 was used to digest out the p53DD fragment of pCE-p53DD vector, following by the vector self-ligation to create the new EBNA-1 expression vector (EBNA-1). To generate a vector that carries only p53DD expression fragment, NheI and AvrII was used to digest out the EBNA-1 fragment of pCE-p53DD vector, following by the vector self-ligation to create the new p53DD-only expression vector (p53DD-only).

b, c, Flow cytometry analysis (**b**), and the quantification of tdTomato negative population % at 48 h post-electroporation with CBE tools (sgRNA and BE3-FNLS), CBE tools with EBNA-1 or p53DD-only or pCE-p53DD (**c**). Unedited H1-SOX2-tdTomato reporter cells was used as gating control, CBE only was used as the editing control, CBE with original pCE-p53DD was used as positive control. n= 3 independent electroporation reactions for each condition. Values presented as mean ± S.D. p values were calculated by ordinary one-way ANOVA.

d, e, Flow cytometry analysis (**d**), and the quantification of tdTomato negative population % at 48 h post-electroporation with PE2 or PE3 tools, PE2 or PE3 tools with EBNA-1 or p53DD-only or pCE-p53DD (**e**). Unedited H1-H2B-tdTomato cell line was used as gating control, PE2 or PE3 only were used as the editing control, PE2 or PE3 with original pCE-p53DD was used as positive control. n= 3 independent electroporation reactions for each condition. Values presented as mean ± S.D. p values were calculated by ordinary one-way ANOVA.

The source data of **c, e** are provided in Source Data file.

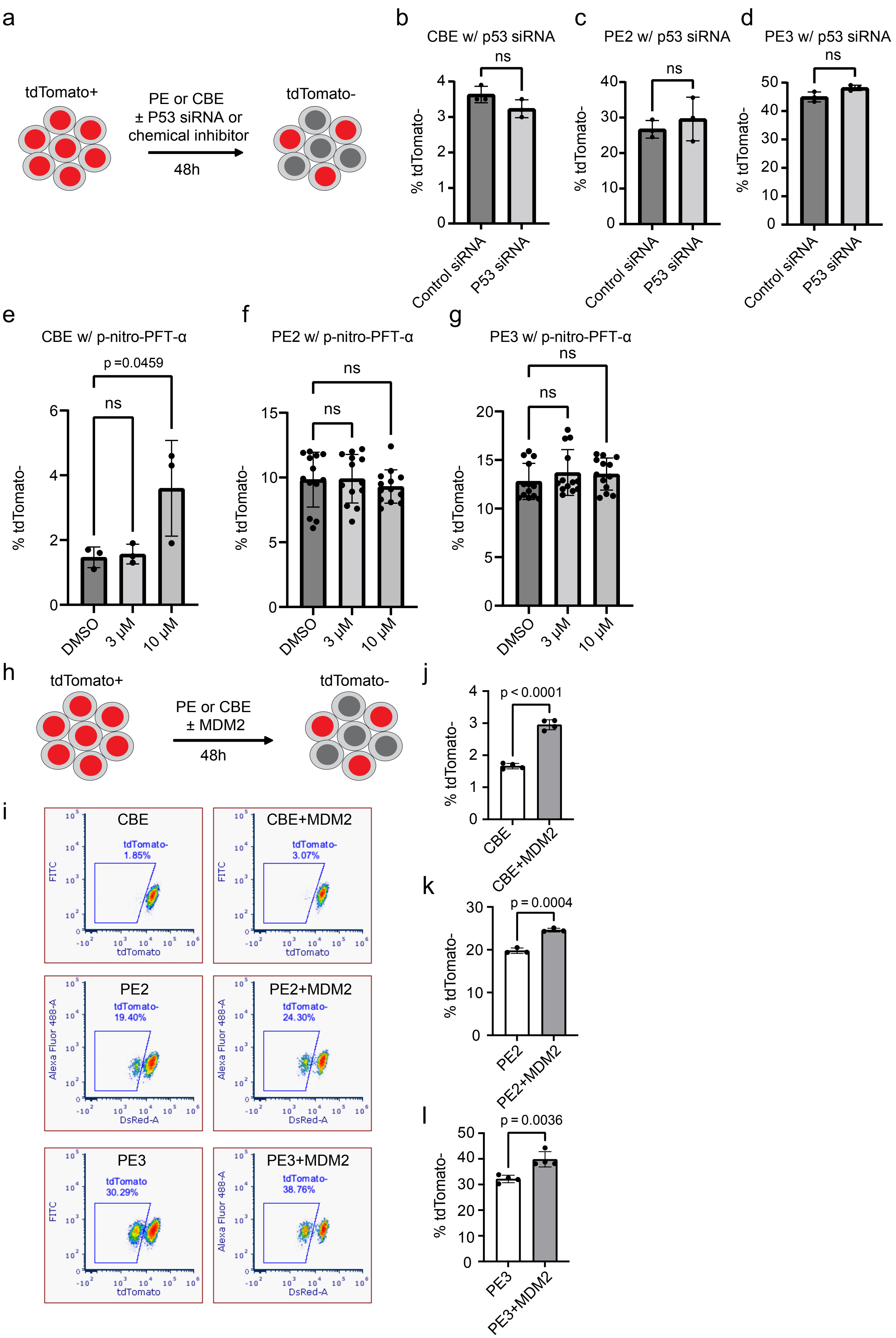
a
tdTomato+
PE or CBE
± P53 siRNA or
chemical inhibitor
48h
tdTomato-
b
CBE w/ p53 siRNA
c
PE2 w/ p53 siRNA
d
PE3 w/ p53 siRNA
ns
% tdTomato-
Control siRNA
P53 siRNA
e
CBE w/ p-nitro-PFT-α
p =0.0459
f
PE2 w/ p-nitro-PFT-α
g
PE3 w/ p-nitro-PFT-α
DMSO
3 μM
10 μM
h
tdTomato+
PE or CBE
± MDM2
48h
tdTomato-
i
CBE
tdTomato-
1.85%
CBE+MDM2
tdTomato-
3.07%
PE2
tdTomato-
19.40%
PE2+MDM2
tdTomato-
24.30%
PE3
tdTomato
30.29%
PE3+MDM2
tdTomato-
38.76%
FITC
Alexa Fluor 488-A
tdTomato
DsRed-A
j
p < 0.0001
CBE
CBE+MDM2
k
p = 0.0004
PE2
PE2+MDM2
l
p = 0.0036
PE3
PE3+MDM2

Supplementary Figure 8. Co-electroporation of MDM2 expression vector, but not p53 siRNA or p53 chemical inhibitor treatment, significantly increases the PE and CBE editing efficiencies.

a. Schematics of evaluating effects of co-electroporation of control or P53 siRNA, or adding p53 chemical inhibitor p-nitro-Pifithrin-α into the PE and CBE editing tool sets respectively, based on "H2B-tdTomato" endogenous reporter assay. tdTomato turn-off rate at 48 h post-electroporation was as the read out. Control siRNA (CST, 6568) or p53 siRNA (CST, 6562) were purchased from Cell Signaling Technology. p-nitro-Pifithrin-α were purchased from Cayman Chemicals (16209).

b-d. The quantification of tdTomato negative population edited in the conditions of CBE (BE3-FNLS) (**b**), PE2 (**c**) or PE3 (**d**) tools, with 100nM control siRNA or 100nM p53 siRNA, in flow cytometry analysis. n= 3 independent experiments for each condition. Values presented as mean ± S.D. p values were calculated by unpaired two-tailed Student's t test. ns. indicates non-significant difference.

e-g. The quantification of tdTomato negative population edited in the conditions of CBE (BE3-FNLS) (**e**), PE2 (**f**) or PE3 (**g**) tools, with indicated doses of p-nitro-Pifithrin-α, in flow cytometry analysis. Upon electroporation, the cells were split to 96-well plates in the medium with of 3 µM or 10 µM of p-nitro-Pifithrin-α, or 1000x DMSO. n=3 wells total for CBE, n= 13 wells total for PE2 or PE3, from 3 independent experiments for each condition. Values presented as mean ± S.D. p values were calculated by ordinary one-way ANOVA. ns. indicates non-significant difference.

h. Schematics of evaluating the effects of co-electroporation of MDM2 expression vector, with the PE or CBE editing tools. tdTomato turn-off rate at 48 h post-electroporation was as the read out.

i-l, Flow cytometry analysis (**i**), and quantification of the tdTomato negative population % at 48 h post-electroporation in the editing conditions of CBE(BE3-FNLS) (**j**), or PE2 (**k**), or PE3 (**l**) tools, with or without MDM2. n= 4 independent experiments for CBE or PE3 editing conditions. n= 3 independent experiments for PE2 editing conditions. Values presented as mean ± S.D. p values were calculated by unpaired two-tailed Student's t test.

The source data of **b-g, j-l** are provided in Source Data file.

Supplementary Fig. 9

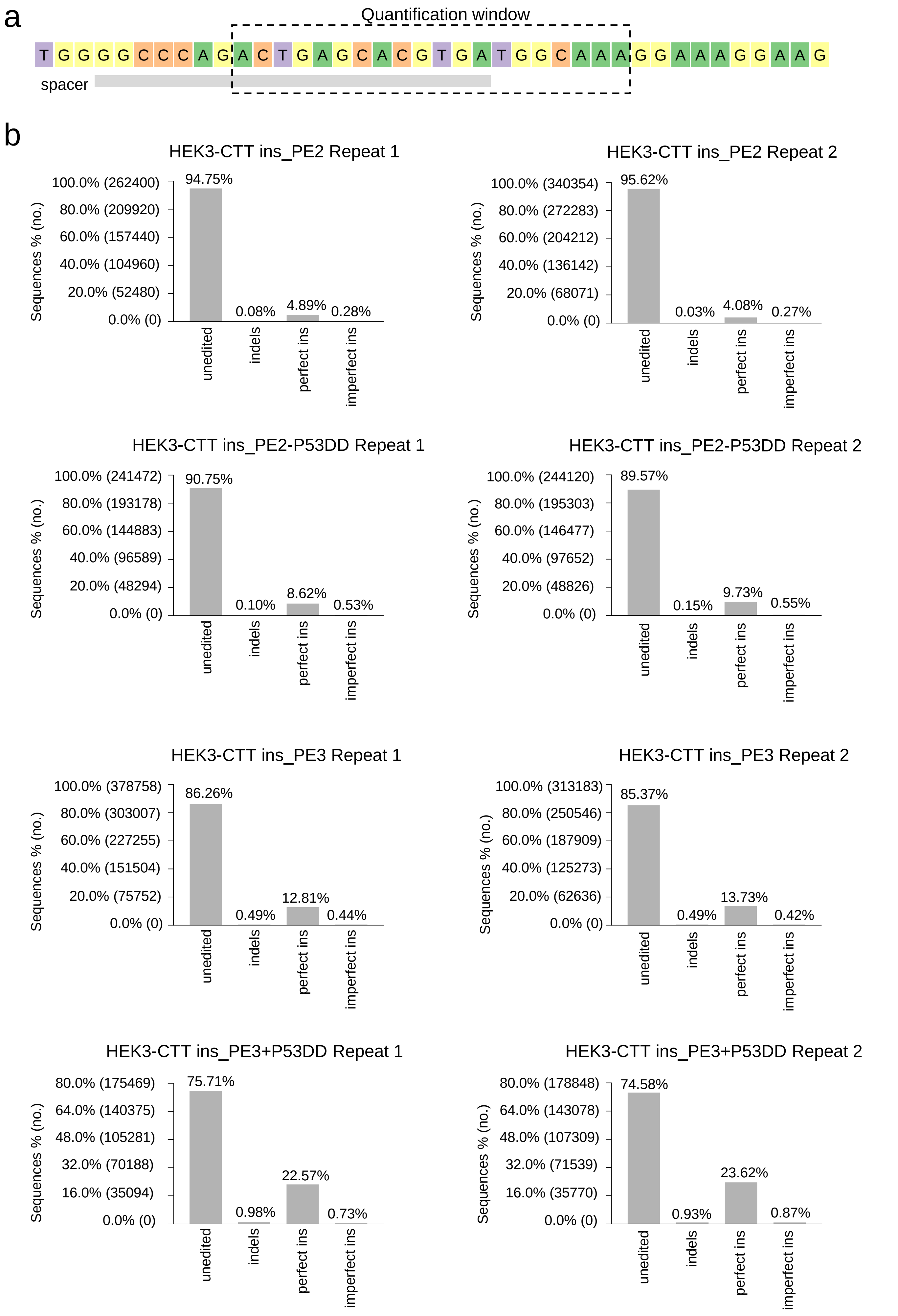

Supplementary Figure 9. Percentage and number of sequence reads were quantified for "CTT" insertion at the HEK3 locus using PE2/3 with or without P53DD. **a,** Alignment and editing frequencies were calculated in a quantification window (in dotted box) which spanning the -10 position to +10 position relative to the nicking site of pegRNA, Grey bar represents the pegRNA spacer. **b,** The non-edited frequency and edited frequency including perfect CTT insertion, imperfect CTT insertion as well as other indels within the reading window were calculated using CRISPResso2 with HDR mode. Two independent experiments were conducted, and the data from individual repeat were shown.

Supplementary Fig. 10

a

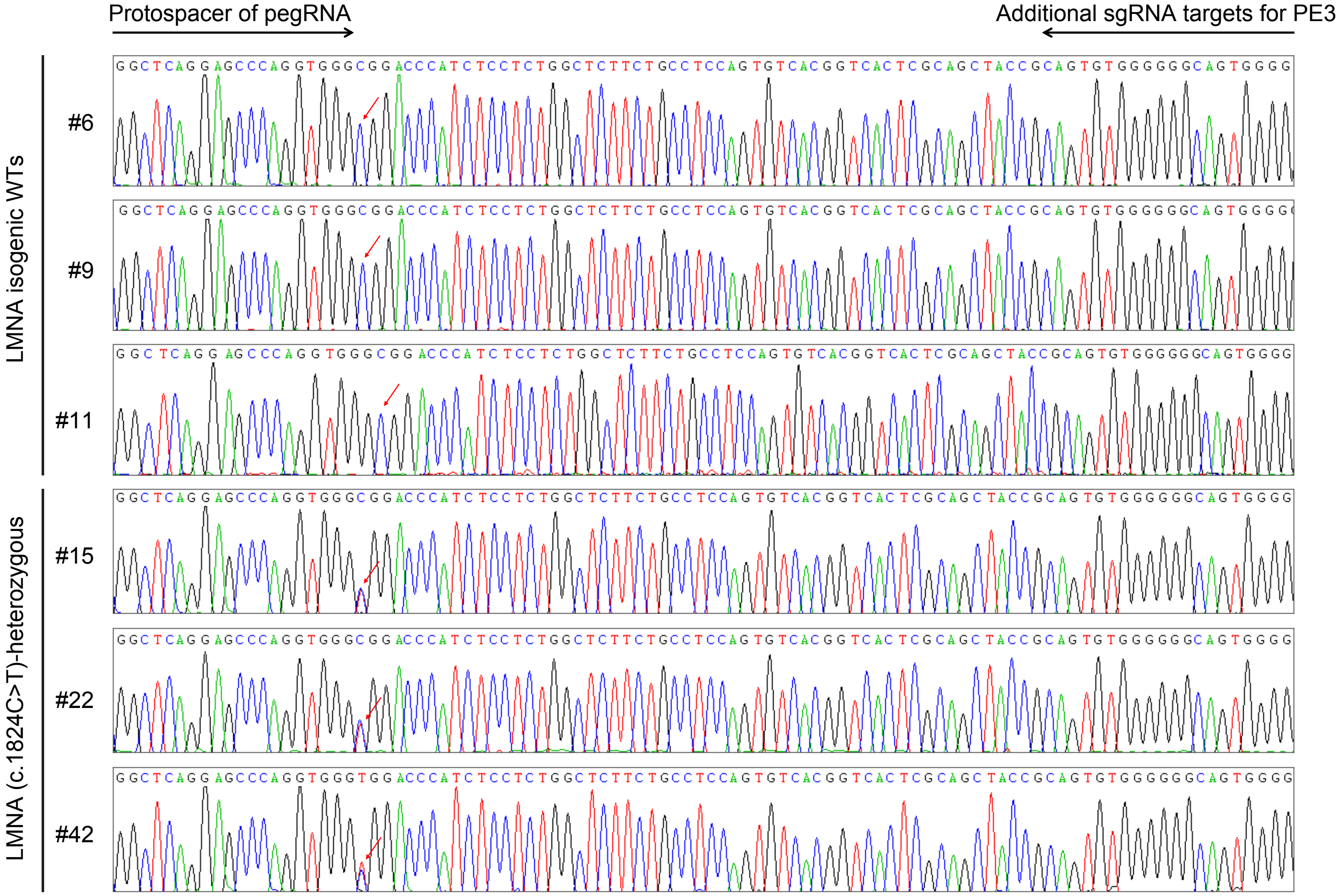

Supplementary Figure 10. Sanger sequencing of single-cell clones of MSK-SRF001-iPSCs with LMNA mutation.

a, Sequences of 3 representative MSK-SRF001 iPSC clones with heterozygous LMNA (c.1824 C>T), and 3 representative isogenic WT clones. Protospacer of the pegRNA and the nicking sgRNA target were noted on top of the sequence. Red arrow indicates the LMNA (c.1824 C>T) mutation. No indels or bystander products were seen in among these single-cell clones.

Supplementary Fig. 11

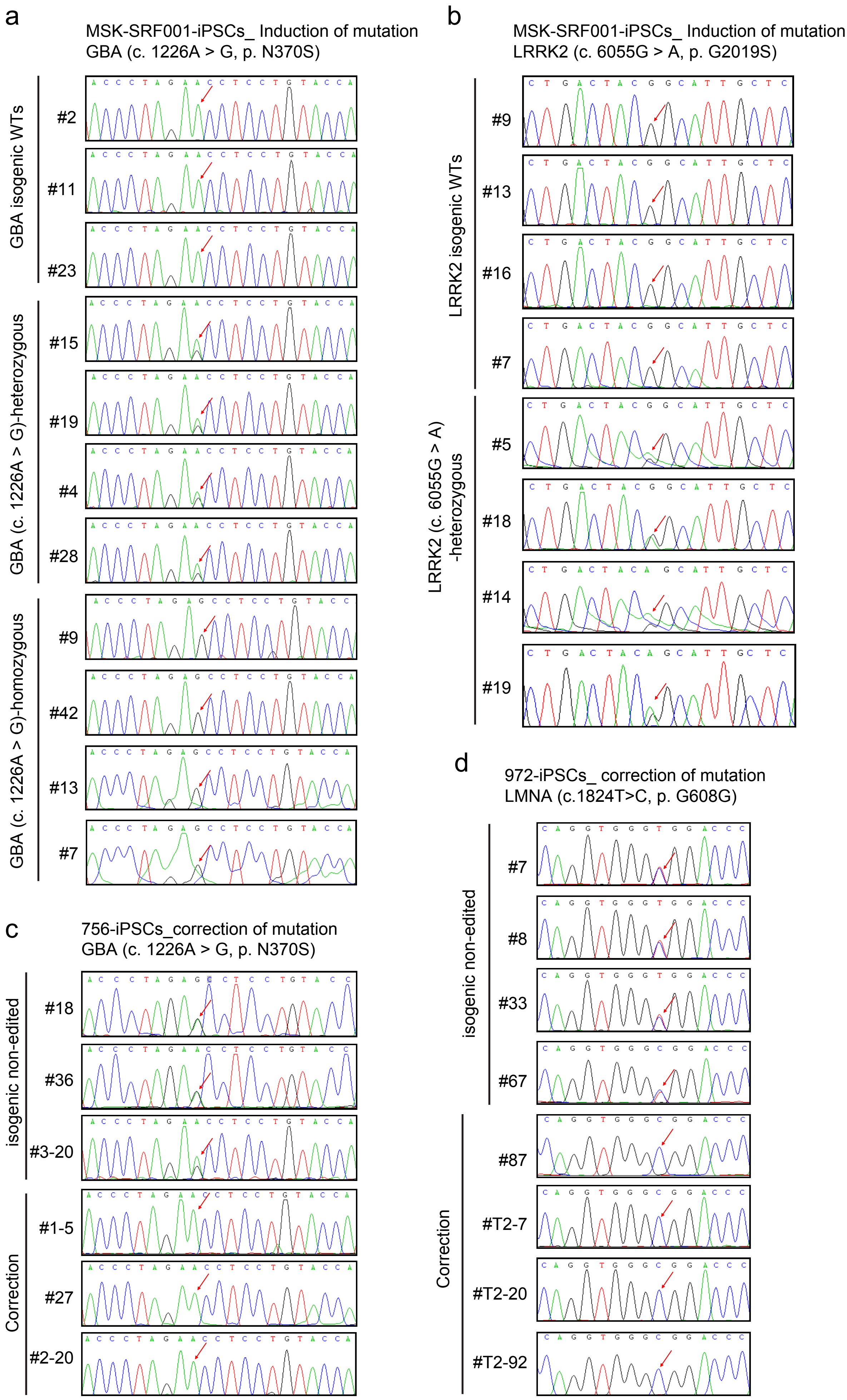

Supplementary Figure 11. Sanger sequencing of single-cell clones edited with PE3+p53DD.

a, Sequences of 4 representative MSK-SRF001 iPSC clones with heterozygous GBA (c. 1226A > G) mutation, 4 representative clones with homozygous GBA (c. 1226A > G) mutation, as well as 3 representative isogenic WT clones. Red arrow indicates the GBA (c. 1226A > G) mutation.

b, Sequences of 4 representative MSK-SRF001 iPSC clones with heterozygous LRRK2 (c. 6055G > A) mutation, and 4 representative isogenic WT clones. Red arrow indicates the LRRK2 (c. 6055G > A) mutation.

c, Sequences of 3 representative 756 iPSC clones with the GBA (c. 1226A > G) mutation corrected, and 3 representative isogenic non-edited clones. Red arrow indicates the GBA (c. 1226A > G) correction.

d, Sequences of 4 representative 972 iPSC clones with the LMNA (c.1824 C>T) mutation corrected, and 4 representative isogenic non-edited clones. Red arrow indicates the LMNA (c.1824 C>T) correction.

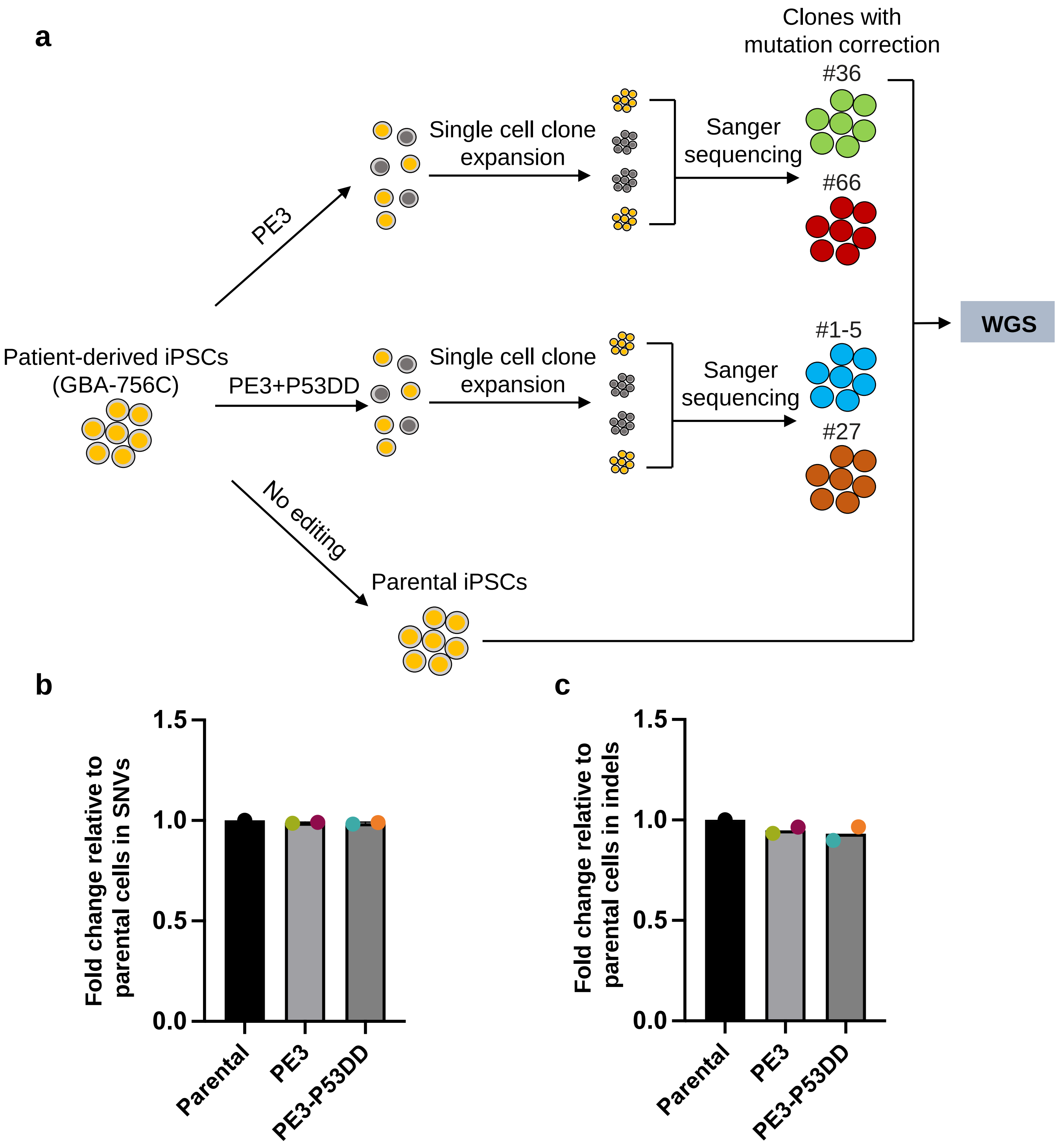
a
Clones with mutation correction
#36
#66
#1-5
#27
PE3
Single cell clone expansion
Sanger sequencing
WGS
Patient-derived iPSCs (GBA-756C)
PE3+P53DD
No editing
Parental iPSCs
b
Fold change relative to parental cells in SNVs
1.5
1.0
0.5
0.0
Parental
PE3
PE3-P53DD
c
Fold change relative to parental cells in indels
1.5
1.0
0.5
0.0
Parental
PE3
PE3-P53DD

Supplementary Figure 12. p53DD did not increase genome-wide off-target effects in patient's mutation corrected single-cell iPSC clones.

a. Schematic overview of experimental design to identify genome-wide SNVs and indels in PE3 or PE3 with p53DD edited single-cell iPSC clones using WGS. Point mutation (c. 1226A > G, p. N370S) in the patient-derived iPS cells was corrected either by PE3 or PE3 with p53DD. Single cell clones were expanded, and the two corrected clones from each condition were determined by sanger sequencing and subjected to WGS. Parental iPSCs were used as the background control for WGS.

b, c Fold change of SNVs (**b**) and insertions and deletions (indels) (**c**) in PE3 or PE3-p53DD corrected clones as compared to the parental iPSCs. n=2 clones with each editing condition.

The source data of **b,c** are provided in Source Data file.

Supplementary Fig. 13

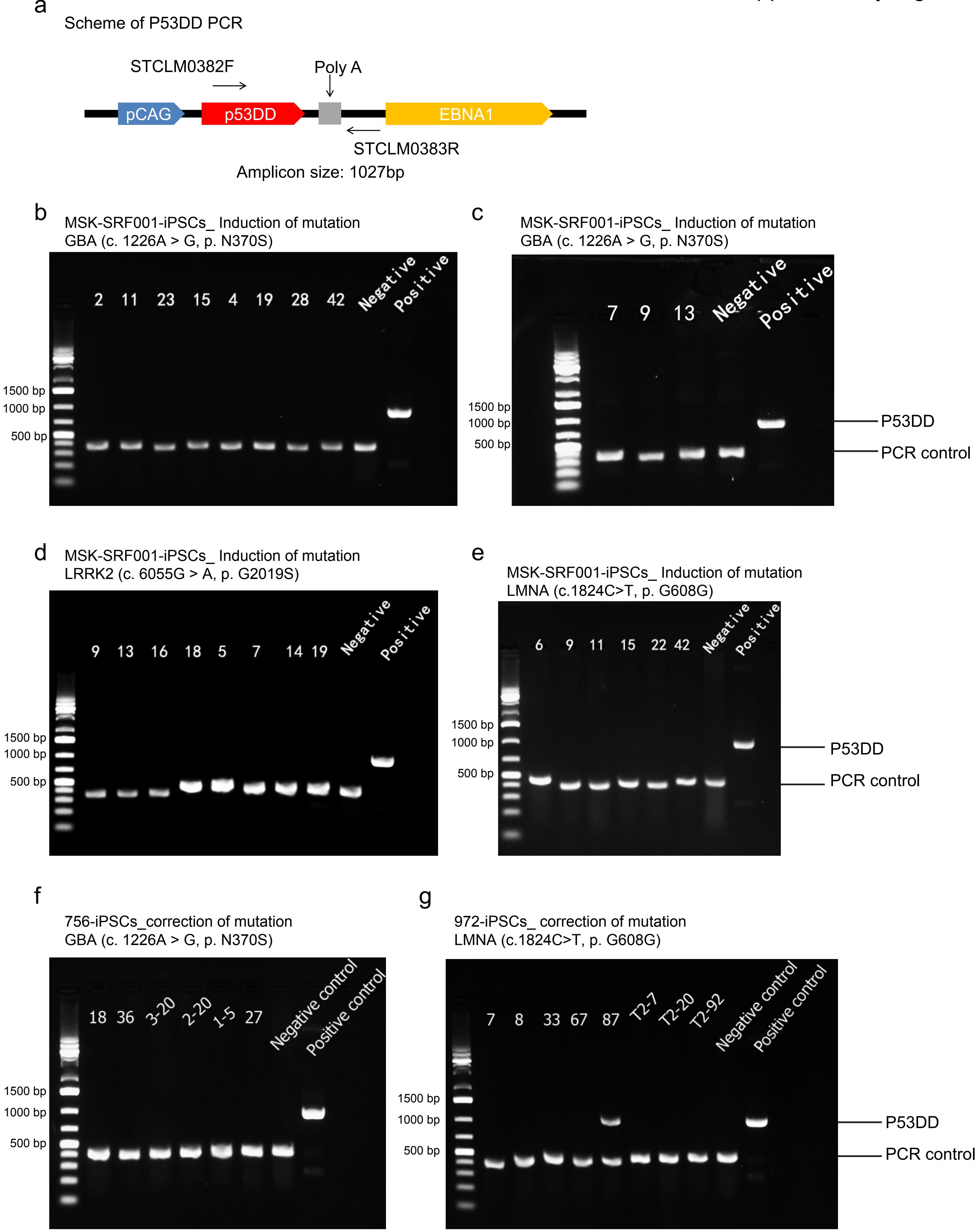

Supplementary Figure 13. PCR results for potential p53DD plasmid integration in the single-cell clones edited with PE3+p53DD.

a, A scheme showing the design of the primer sets used to amplify a part of the p53DD construct as detection for potential p53DD plasmid integration.

b-g, p53DD PCR results. PCR results of in MSK-SRF001-iPSC clones with induced mutations, including GBA (c. 1226A > G, p. N370S) (**b**,**c**), LRRK2 (c. 6055G > A, p. G2019S) (**d**), and LMNA (c.1824 C>T; p.G608G) (**e**). PCR results of corrected patients' iPSC clones, including GBA (c. 1226A > G, p. N370S) correction in 756-iPSCs (**f**), and LMNA (c.1824 C>T; p.G608G) correction in 972-iPSCs (**g**). PCR for LMNA were used as the PCR reaction control for evaluating the quality of the genomic DNA and the PCR reactions (PCR size: 414 bp). A positive control (p53DD plasmid), and a negative control (cells without p53DD electroporation) were used at the same time for each experiment. PCR analysis detected one clone (972-iPSC-LMNA correction #87) out of total 39 had p53DD integration. The other clones were negative according to PCR results. PCR were performed once from genome DNA of the sanger sequencing confirmed clones.

Supplementary Fig. 14

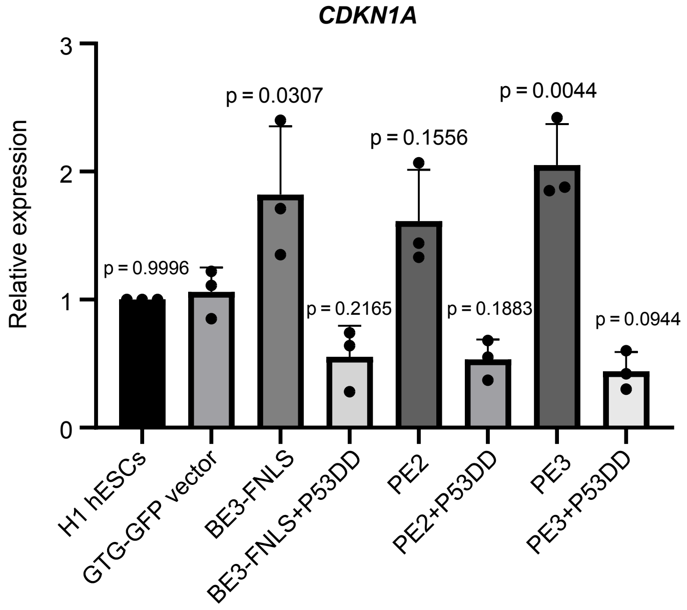

Supplementary Figure 14. qPCR quantification of CDKN1A expression.

The CDKN1A mRNA expression was significantly increased in CBE and PE3 edited, and moderately increase in PE2 edited cells compare to GTG-GFP vector alone electroporated cells. With additional of p53DD in each editing tool, the CDKN1A mRNA expression was downregulated close to the control level. The relative expression value of each condition was normalized to non-electroporated H1 hESCs. n= 3 independent electroporation reactions for each editing condition. Values presented as mean ± S.D. p values were calculated by ordinary one-way ANOVA test for multiple comparisons to a control group (GTG-GFP vector alone electroporation samples). The source data are provided in Source Data file.

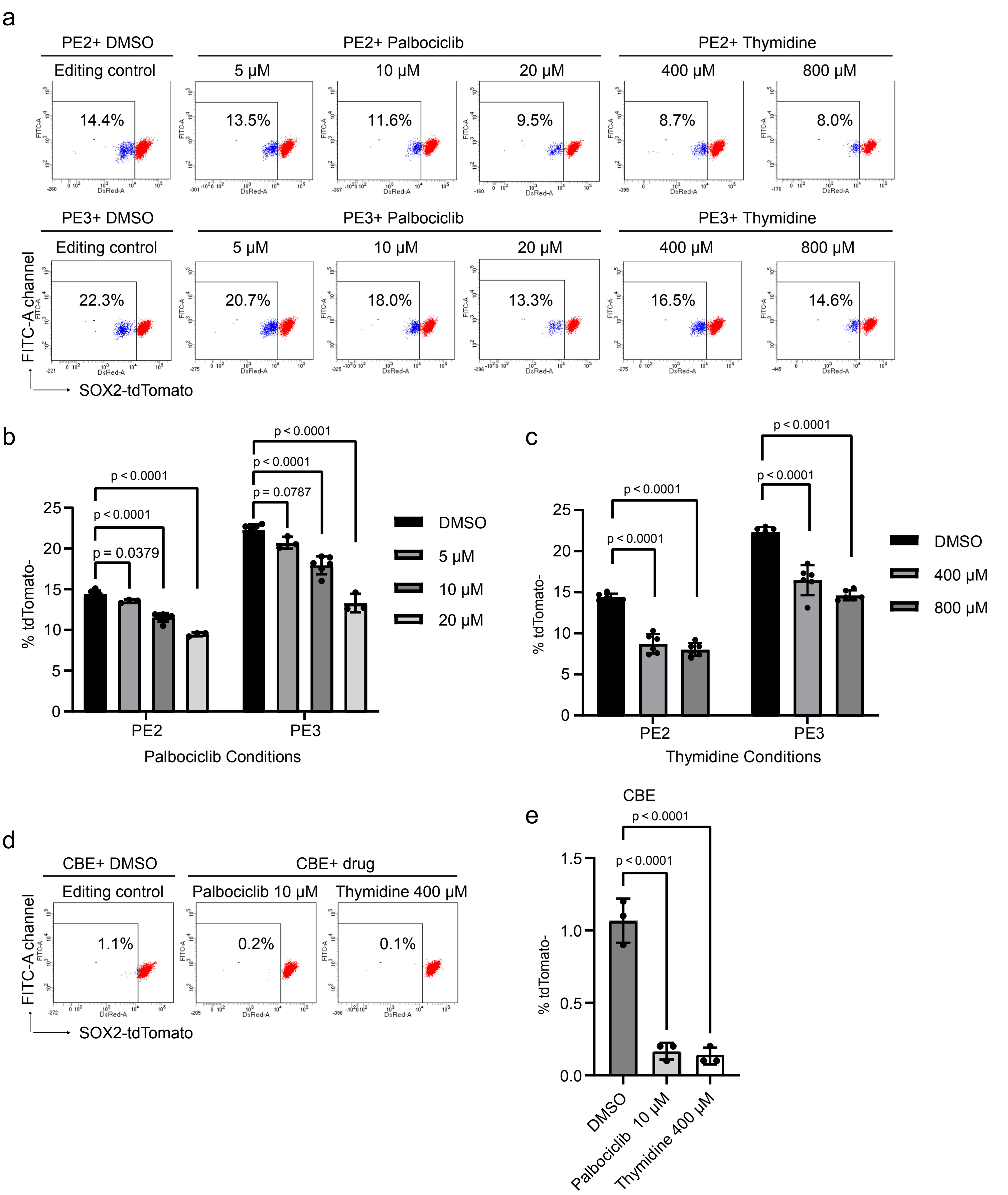
a
PE2+ DMSO
Editing control
PE2+ Palbociclib
5 μM
10 μM
20 μM
PE2+ Thymidine
400 μM
800 μM
14.4%
13.5%
11.6%
9.5%
8.7%
8.0%
PE3+ DMSO
PE3+ Palbociclib
PE3+ Thymidine
22.3%
20.7%
18.0%
13.3%
16.5%
14.6%
FITC-A channel
SOX2-tdTomato
b
% tdTomato-
p = 0.0379
p < 0.0001
p < 0.0001
p = 0.0787
p < 0.0001
p < 0.0001
PE2
PE3
Palbociclib Conditions
DMSO
5 μM
10 μM
20 μM
c
% tdTomato-
p < 0.0001
p < 0.0001
p < 0.0001
p < 0.0001
Thymidine Conditions
DMSO
400 μM
800 μM
d
CBE+ DMSO
Editing control
CBE+ drug
Palbociclib 10 μM
Thymidine 400 μM
1.1%
0.2%
0.1%
FITC-A channel
SOX2-tdTomato
e
CBE
p < 0.0001
p < 0.0001
% tdTomato-
DMSO
Palbociclib 10 μM
Thymidine 400 μM

Supplementary Figure 15. CDK4-6 inhibitor (Palbociclib), or a DNA synthesis inhibitor (Thymidine) treatment decreased PE and CBE editing efficiencies in hPSCs.

a-e, Flow cytometry analysis (**a**), and the quantification of tdTomato negative population % at 48 h post-electroporation with PE2 (**a**,**b**) or PE3 (**a**,**c**) or CBE (BE3-FNLS) (**d,e**) editing tools, with or without indicated doses of Palbociclib and Thymidine. Palbociclib were purchased from Sigma (T1895). Thymidine were purchased from Sigma (PZ0383). Upon electroporation, the cells were split to 96-well plates in the medium with of 5 µM or 10 µM or 20 µM of Palbociclib, 400 µM or 800 µM of Thymidine, or 1000x DMSO. For PE2 and PE3 editing experiments, n=3 wells total for 5 µM Palbociclib or 20 µM Palbociclib treatment; n= 6 wells total for all other condition treatment, from 3 independent experiments. For CBE editing experiments, n= 3 wells total for each condition from 3 independent experiments. Unedited H1-SOX2-tdTomato reporter cells was used as gating control, PE2 or PE3 or CBE with DMSO condition was used as the editing control. Values presented as mean ± S.D. p values were calculated by ordinary one-way ANOVA.

The source data of **b, c, e** are provided in Source Data file.

Supplementary Fig. 16

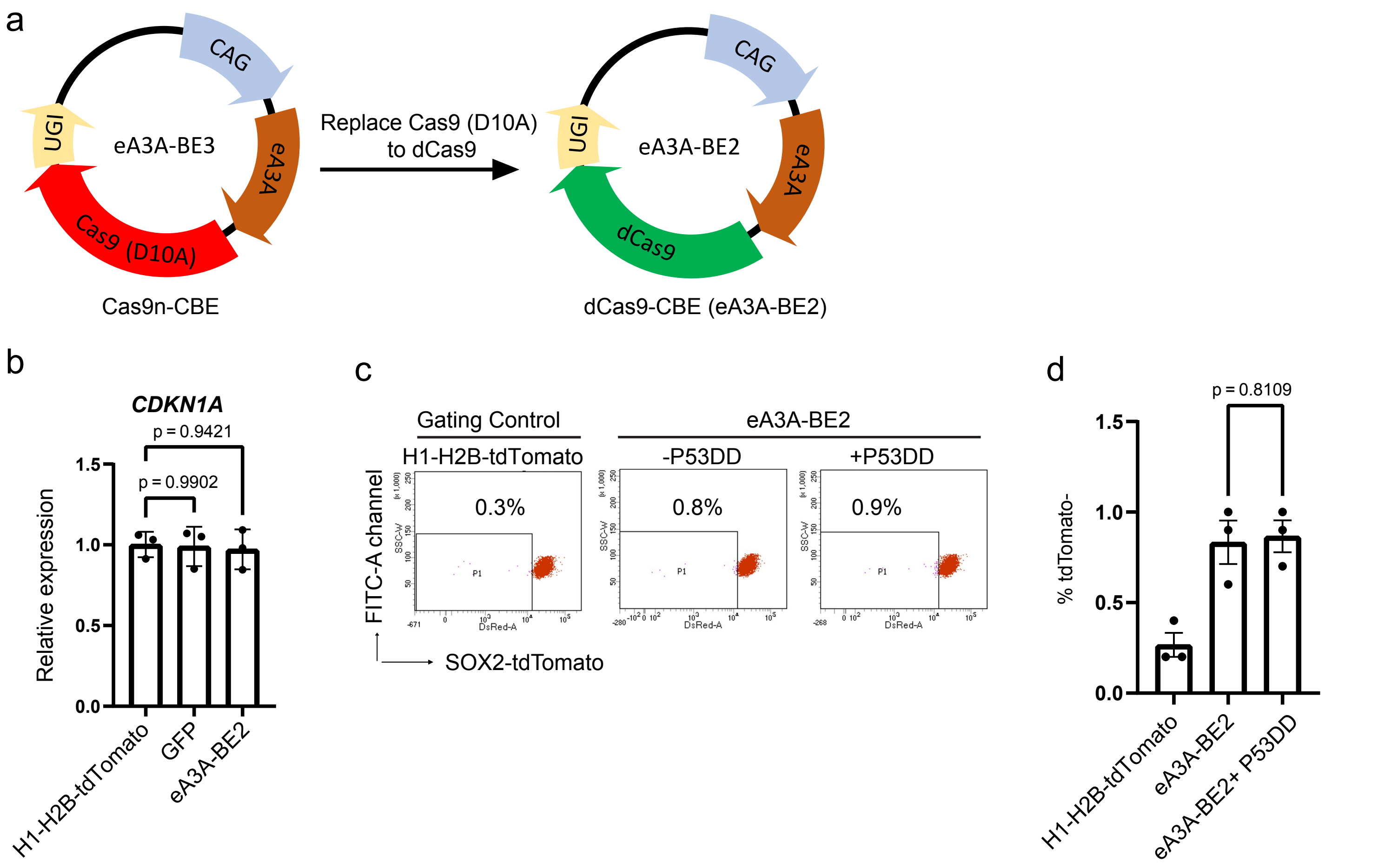

Supplementary Figure 16. p53DD did not increase the editing efficiency of dCas9-CBE (eA3A-BE2) in hPSCs.

a, A scheme showing the re-construction of the CBE vector (eA3A-BE3) by replacing the Cas9n to dCas9 to create dCas9-CBE (eA3A-BE2) editing tool.

b, qPCR quantification of CDKN1A (p21 gene) expression in GFP expression vector electroporated cells or eA3A-BE2 edited cells. RNA sample were collected at 48 h post-electroporation. The relative expression value of each condition was normalized to non-electroporated H1-H2B-tdTomato reporter hESCs. n= 3 independent electroporation reactions for each editing condition. Values presented as mean ± S.D. p values were calculated by ordinary one-way ANOVA test, and indicates non-significant difference.

c, d, Flow cytometry analysis (**c**), and the quantification of tdTomato negative population % at 48 h post-electroporation with eA3A-BE2) editing tool, with or without p53DD(**d**). n= 3 independent electroporation reactions for each condition. Values presented as mean ± S.D. p values were calculated by ordinary one-way ANOVA, and indicates non-significant difference.

The source data of **b, d** are provided in Source Data file.

Supplementary Fig. 17

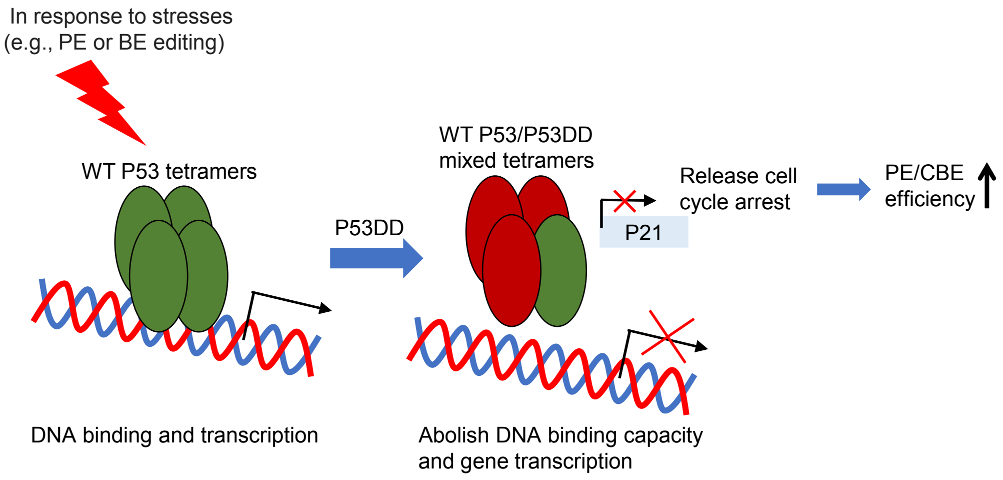

Supplementary Figure 17. Proposed Mechanism of p53DD to enhance PE and CBE editing efficiency.

In response to stresses, p53 functions as a tetramer to regulate downstream gene transcription[1]. The p53DD acts as the dominant negative format of WT p53, which carries the tetramer domain and C-terminal domain, but lacks of the DNA-binding domain[2]. p53DD protein is capable of forming mixed tetramers with WT p53 proteins, and prevent the binding of the p53 tetramer to DNA[3, 4] and the gene transcription (e.g. p21 gene expression). The consequent release of potential cell cycle arrest may explain how p53DD improve PE and CBE editing efficiency in hPSCs.

Supplementary Fig. 18

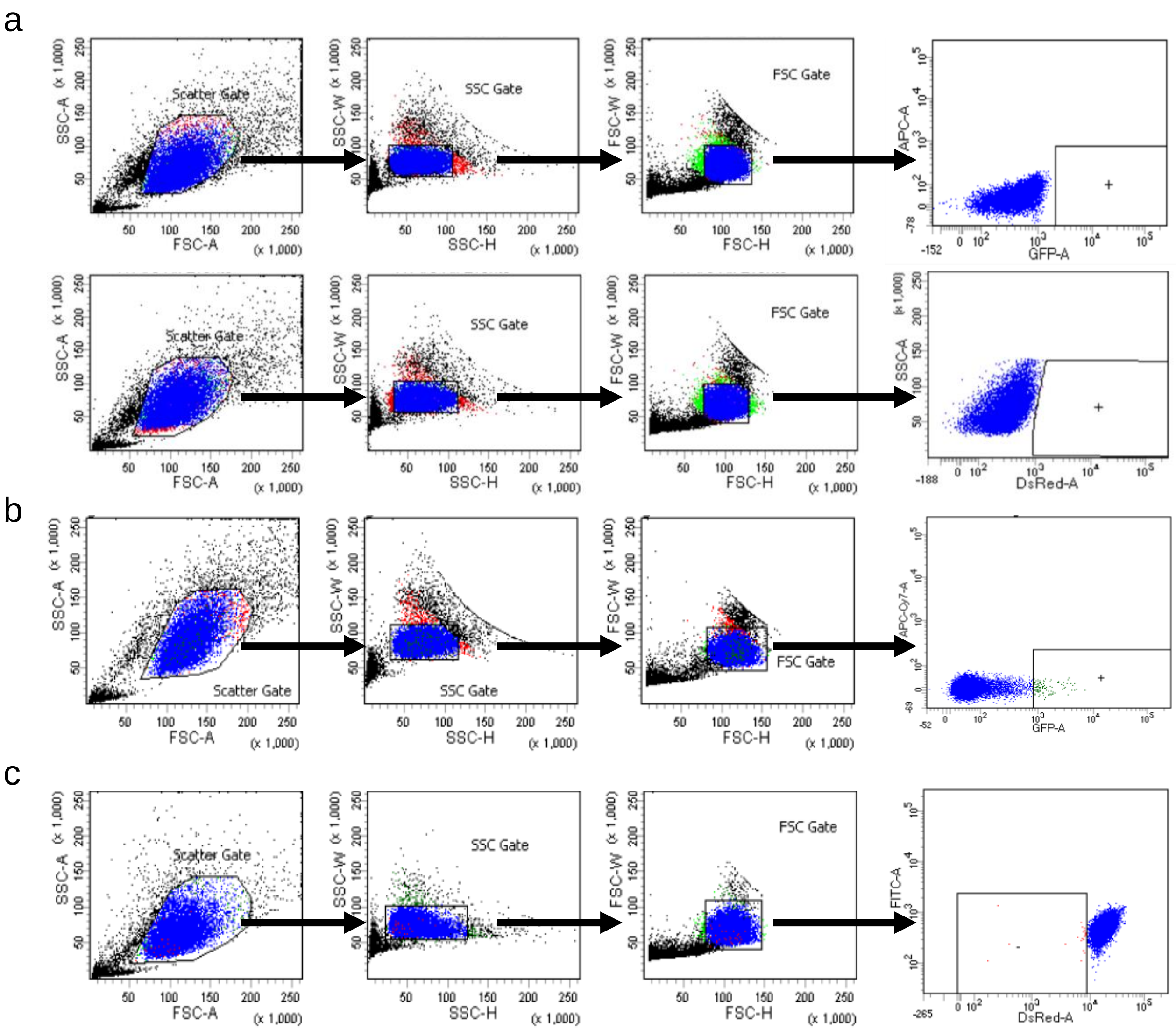

Supplementary Figure 18. Example FACS gating strategy used for reporter assays.

Live cells were selected (forward scatter area (FSC-A) versus side scatter area (SSC A)), followed by selection of single cells ((forward scatter width (FSC-W) versus height (FSC-H) and side scatter width (SSC-W) versus height (SSC-H)).

a. For measuring HDR efficiency using a hESCs knock-in reporter, APC-A channel (non-staining, Y- axis) and the GFP-A channel (GFP, X- axis) was used to detect POU5F1-GFP positive and negative cell population. The FITC-A channel (non-staining, Y- axis) and the DsRed channel (SOX2-tdTomato, X- axis) was used to detect SOX2-tdTomato positive and negative cell population. The H1 hESCs were used as gating controls. Data presented in Supplementary Fig. 1.

b. For measuring PE and CBE editing efficiency using a "GTG-GFP" reporter vector, APC-Cy7-A channel (non-staining, Y- axis) and the GFP-A channel (GFP, X- axis) was used to detect GFP positive and negative cell population. "GTG-GFP" vector electroporated cells were used as gating controls. Data presented in Supplementary Fig. 2 and 3.

c. For measuring PE and CBE editing efficiency using a "H1-SOX2-H2B-tdTomato" reporter line, FITC-A channel (non-staining, Y- axis) and the DsRed channel (SOX2-tdTomato, X- axis) was used to detect SOX2-tdTomato positive and negative cell population. The H1-SOX2-H2B-tdTomato hESCs were used as gating controls. Data presented in Fig. 1, Supplementary Fig. 6, Supplementary Fig. 7, Supplementary Fig. 8, Supplementary Fig. 15, Supplementary Fig. 16.

Supplementary Table 1. Isogenic diseases' iPSC clones generated by PE3+P53DD, and the characterization.

Title	Parental line	clone #	Sanger sequence confirmed	P53DD PCR	Pluripotent maker expression
Induction of mutation GBA (c. 1226A > G, p. N370S)	MSK-SRF001-iPSCs	2	WT	√	√
		11	WT	√	√
		23	WT	√	√
		15	Heter-1226A > G	√	√
		4	Heter-1226A > G	√	√
		19	Heter-1226A > G	√	√
		28	Heter-1226A > G	√	√
		42	Homo-1226A > G	√	√
		7	Homo-1226A > G	√	√
		9	Homo-1226A > G	√	√
		13	Homo-1226A > G	√	√
Correction of mutation GBA (c. 1226A > G, p. N370S)	756-iPSCs	18	non-edit	√	√
		36	non-edit	√	√
		3-20	non-edit	√	√
		2-20	correction	√	√
		1-5	correction	√	√
		27	correction	√	√
Induction of Mutation: LMNA (c.1824C>T, p. G608G)	MSK-SRF001-iPSCs	6	WT	√	√
		9	WT	√	√
		11	WT	√	√
		15	Heter-1824C>T	√	√
		22	Heter-1824C>T	√	√
		42	Heter-1824C>T	√	√
Correction of Mutation: LMNA (c.1824C>T, p. G608G)	972-iPSCs	7	Non-edit	√	√
		8	Non-edit	√	√
		33	Non-edit	√	√
		67	Non-edit	√	√
		87	Correction	detected	N/A
		T2-7	Correction	√	√

		T2-20	Correction	√	√
		T2-92	Correction	√	√
Induction of mutation LRRK2 (c. 6055G > A, p. G2019S)	MSK-SRF001-iPSCs	9	WT	√	√
		13	WT	√	√
		16	WT	√	√
		7	WT	√	√
		5	Heter-G2019S	√	√
		18	Heter-G2019S	√	√
		14	Heter-G2019S	√	√
		19	Heter-G2019S	√	√

Supplementary Table 2. Summary of sgRNA target sequences used in CRIPSR/HDR, CBE, or PE3 editing related experiments. Sequences are shown in 5' to 3' orientation.

SgRNA construct	sgRNA target	Purpose	Used in experiments
PX330-POU5F1-GFP	TCTCCCATGCATTCAAACTG	CRIPSR/HDR	Supplementary Fig.1b, c
PX330-SOX2-tdTomato	CCTCTCACACATGTGAGGGC	CRIPSR/HDR	Supplementary Fig.1e, f
Lsg-GTG-GFP	ACCACAATTATATATGCAGG	CBE	Supplementary Fig.2d, e, g; Supplementary Fig.3b, d
Lsg-H2B-tdTomato	AAGCAGGTCCACCCTGACAC	CBE	Fig.1d, e, j; Supplementary Fig.4c, 6b-c, 7b-c,8b,8e,8i-j, 15d-e, 16b-d
Lsg-PE3-GTG-GFP	CGCCGGACACGCTGAACTTG	PE3	Supplementary Fig.2d, e, f; Supplementary Fig.3b, c
Lsg-H2B-nick	CCTGCTGCTGCCTGGGGAGT	PE3	Fig.1f, g, h, k. Supplementary Fig.6d-f, 7d-e, 8c-d, 8f-g,8i, 8k-l,15a-c

Lsg-HEK3-nick	GTCAACCAGTATCCCGGTGC	PE3	Fig.2b, c, d
Lsg-SERPING1-nick	CTTGGAGAGTCATTCAACAG	PE3	Fig.2e
Lsg-GBA-1+2	ACCCTTACCTACACTCTCTG	PE3	Fig.3b, e, g
Lsg-LRRK2-1	GACAGACCTGATCACCTACC	PE3	Fig.3c, g
Lsg-LMNA-1+2	CCCCACTGCCCCCCACACTG	PE3	Fig.3d, f, g

Supplementary Table 3. Summary of pegRNAs and nicking sgRNA sequences used in PE editing replated experiments. 3' extension of pegRNA contains PBS and RT template sequences. Sequences are shown in 5' to 3' orientation.

PegRNA construct	Spacer sequence	3' extension sequence	Used in experiments
pegRNA-GTG-GFP-24	CCCACCTGCATA TATAATTG	TTGCTCACCATAATTATATA TGCAGGTG	Supplementary Fig.2d, e, f; Supplementary Fig.3b, c
pegRNA-H2B-stop	CTCACCATGGTG GCGACCGG	GGATCCACCGTGAGTCGC CACCATGG	Fig.1f, g, h, k
PegRNA-HEK3-"CTT" insertion	GGCCCAGACTGA GCACGTGA	TGCCATCAAAGCGTGCTCA GTCTG	Fig.2b
PegRNA-HEK3-"Loxp" insertion	GGCCCAGACTGA GCACGTGA	TTTGCCATCAATAACTTCG TATAATGTATGCTATACGA AGTTATCGTGCTCAGTCTG	Fig.2c
PegRNA-HEK3-"GT" deletion	GGCCCAGACTGA GCACGTGA	TCCTTTGCCATCGTGCTCA GTCTG	Fig.2d
PegRNA-SERPING1 (c.351delC)	GAAGGACCCAGT AGTGGGCT	TCCTACCCAGCCACTACTG GGTC	Fig.2e
pegRNA-GBA-10	AGCCGACCACAT GGTACAGG	TTACCCTAGAGCCTCCTGT ACCATGTGGTC	Fig.3b, g
pegRNA-GBA-4	AGCCGACCACAT GGTACAGG	TTACCCTAGAACCTCCTGT ACCATGTGGTC	Fig.3e, g
pegRNA-LRRK2-3	ATTGCAAAGATTG CTGACTA	AGCAATGCTGTAGTCAGCA ATCTTTGC	Fig.3c, g
pegRNA LMNA-1	GGCTCAGGAGCC CAGGTGGG	ATGGGTCCACCCACCTGG GCTCCTG	Fig.3d, g
pegRNA-LMNA-CR-2-5	GCCGACAAGGCA TCTGCCAG	AGATGGGTCCGCCCACCT GGGCTCCTGAGCCGCTGG CAGATGCCTTGT	Fig.3f, g

Supplementary Table 4. Summary of PCR primers used in PCR for Miseq or sanger sequencing. Sequences are shown in 5' to 3' orientation.

Purpose	PCR-Forward	PCR-Reverse
H2B-tdTomato-CBE editing	ATGCCAGAGCCAGCGAAGTCT	CGCTGGTGTACTTGGTGATGG
H2B-tdTomato-PE editing	ACACCAGCGCTAAGGATCCAC	CACCGTGTCCCAGGCGAAGGGCAGG
HEK3-CTT or Loxp insertion	CTTGGCATGAGAAACCTTGG	TCCCTCCTCTCCTGGTGAG
GBA mutation induction or correction	CCTTGAGATGCCTGGATCTTC	CGACAAAGTTACGCACCCAAT
LMNA mutation induction or correction	TGGGCACAGAACCACACCTTC	AGACAAAGCAGAGACAACTCACCT
LRRK2 mutation induction	TAAGGGACAAAGTGAGCACAG	CACATCTGAGGTCAGTGGTTATC
pegRNA-off-1	AGGATCAAAGCCCCAAAAGC	AGTCAGATGCCACAGAGACC
pegRNA-off-2	GCCAAAGGATCAACAGCCAA	GACGACTGTATCACTCCCGT
pegRNA-off-3	GGACACTAGAGAGCAGGCAT	CATCCCCTCACACACTGACT
nicking sgRNA-off-1	TATGCTGCTTCCTCCACGTT	AGCTCTGCGTCTTAGAACCA

Supplemental Table 5- Indel frequency analysis at potential off-target loci in PE3 with or without P53DD editing for "CTT" insertion at *HEK3* locus. Related to Fig 2b.

Spacer (5'-3')	PAM	Chromosome	Position	Mismatches (bp)	Off-target
GGCTCAGACTGAGCACCTGA	GAG	chr2	239105064	2	pegRNA-off-1
CACCCAGACTGAGCACGTGC	TGG	chr15	79457589	3	pegRNA-off-2
GAGCCAGAATGAGCACGTGA	GGG	chr10	129794857	3	pegRNA-off-3
GTCCACCAGGAGCCCGGTGC	TAG	chr19	48607506	3	nicking sgRNA-off-1

Supplemental Table 5a: Sequences with guide-target mismatch <=3, compared to the pegRNA or nicking sgRNA targets. Mismatch targeting sites were found using Benchling. Red letters indicate the mismatches.

Off target	Editing Condition	Experiment 1	Experiment 2	Experiment 3	Average
pegRNA-off-1	PE3	0	0	0	0
	PE3+p53DD	0	0	0	0
pegRNA-off-2	PE3	0	0	0	0
	PE3+p53DD	0	0	0	0
pegRNA-off-3	PE3	0.08%	0.06%	0.18%	0.11%
	PE3+p53DD	0.13%	0.14%	0.13%	0.13%
nicking sgRNA-off-1	PE3	0	0	0	0
	PE3+p53DD	0	0	0	0

Supplemental Table 5b: The percentage of indel frequency at the cutting site of each off-target loci. Indel frequency were detected using PCR and Miseq. PCR primers were listed in supplemental table 4.

References

1. Friedman PN, Chen X, Bargonetti J, Prives C. The p53 protein is an unusually shaped tetramer that binds directly to DNA. *Proceedings of the National Academy of Sciences of the United States of America* **90**, 3319-3323 (1993).

2. Shaulian E, Haviv I, Shaul Y, Oren M. Transcriptional repression by the C-terminal domain of p53. *Oncogene* **10**, 671-680 (1995).

3. Gencel-Augusto J, Lozano G. p53 tetramerization: at the center of the dominant-negative effect of mutant p53. *Genes Dev* **34**, 1128-1146 (2020).

4. Midgley CA, Lane DP. p53 protein stability in tumour cells is not determined by mutation but is dependent on Mdm2 binding. *Oncogene* **15**, 1179-1189 (1997).
