# Supplementary material for: Transient inhibition of p53 enhances prime editing and cytosine base-editing efficiencies in human pluripotent stem cells
Source: Nat Commun. 2022 Oct 27;13:6354. doi: 10.1038/s41467-022-34045-7 (PMC9613702; doi:10.1038/s41467-022-34045-7)
Supplement: Supplementary file 3 — Reporting Summary [file 41467_2022_34045_MOESM3_ESM.pdf]

nature portfolio

Corresponding author(s): Lorenz Studer and Ting Zhou

Last updated by author(s): 25.09.22

Reporting Summary

Nature Portfolio wishes to improve the reproducibility of the work that we publish. This form provides structure for consistency and transparency in reporting. For further information on Nature Portfolio policies, see our Editorial Policies and the Editorial Policy Checklist.

Statistics

For all statistical analyses, confirm that the following items are present in the figure legend, table legend, main text, or Methods section.

n/a	Confirmed	
☐	☒	The exact sample size (*n*) for each experimental group/condition, given as a discrete number and unit of measurement
☒	☐	A statement on whether measurements were taken from distinct samples or whether the same sample was measured repeatedly
☐	☒	The statistical test(s) used AND whether they are one- or two-sided<br>*Only common tests should be described solely by name; describe more complex techniques in the Methods section.*
☒	☐	A description of all covariates tested
☒	☐	A description of any assumptions or corrections, such as tests of normality and adjustment for multiple comparisons
☒	☐	A full description of the statistical parameters including central tendency (e.g. means) or other basic estimates (e.g. regression coefficient) AND variation (e.g. standard deviation) or associated estimates of uncertainty (e.g. confidence intervals)
☐	☒	For null hypothesis testing, the test statistic (e.g. *F*, *t*, *r*) with confidence intervals, effect sizes, degrees of freedom and *P* value noted<br>*Give P values as exact values whenever suitable.*
☒	☐	For Bayesian analysis, information on the choice of priors and Markov chain Monte Carlo settings
☒	☐	For hierarchical and complex designs, identification of the appropriate level for tests and full reporting of outcomes
☒	☐	Estimates of effect sizes (e.g. Cohen's *d*, Pearson's *r*), indicating how they were calculated

Our web collection on statistics for biologists contains articles on many of the points above.

Software and code

Policy information about availability of computer code

Data collection	Flow cytometry samples were run using BD FACSAria III instrument. Sanger sequencing data was collected by submitting PCR product to Eton Biosciences. Miseq data was collected by submitting purified PCR product to Genewiz for Amplicon sequencing (Amplicon EZ service, Illumina MiSeq system). All graphs were produced in GraphPad Prism (9.2.0).<br>For RNA-seq, RNA library was prepared using NEBNext Ultra RNA Library Prep Kit (New England Biolabs) for Illumina following the manufacturer's instructions and underwent 2X150-bp sequenced on an Illumina HiSeq System by GENEWIZ.<br>For WGS, DNA libraries were prepared with standard Illumina protocols and were sequenced with Illumina Hi-seq 4000 platform using 2X150 bp paired-end configuration by GENEWIZ. Base calls and quality scores were stored in.bcl files which were then converted into fastq files by using HiSeq Analysis Software (HAS) v2.2 suite. De-multiplexing was performed according to barcodes for the samples.
Data analysis	Flow cytometry data was analyzed using BD FACSDiva Version 8.0. Miseq data was analysis by alignment of amplicon sequences to a reference sequence using open web resource CRISPResso2: (https://crispresso.pinellolab.partners.org/submission).<br>RNA-seq data alignment analysis was performed by MSK Bioinformatic Core. Fastq files were mapped to the targeted genome using the STAR aligner version 2.5.0a (Linux_x86_64). Two-pass alignment was used in which the reads are mapped twice. PICARD tools were used for post-processing of the output SAM files to add read groups and convert SAM files to a compressed BAM format. RNA base-editing variants were called using standard GATK. To quantify C-to-U changes, variants were further filtered by comparison with unedited control sample as follows: (1) The control sample has to be either a "C" or a "G". (2) The base frequency has to be >99%; ie of all the reads mapping to the site only 1% can be an "error". (3) The mutation has to be C>T or G>A. (4) The control sample has to have a depth more than 3.<br>For WGS data analysis, sequence reads were mapped against human reference genome (NCBI GRCh38) using Issac Aligner and the identified duplicate reads were removed from downstream analysis. The Issac Variant Caller was applied to detect single nucleotide variants (SNVs) and small indels up to 50 bp in the samples compared to the reference genome sequence. For both CBE and PE edited samples, eight additional filters were performed to obtain high-confidece variant calls: 1. IndelConflict to remove locus which in region with conflicting indel calls; 2.

SiteConflict to exclude sites with an overlapping indel call; 3. LowGOX to filter out locus with GQX (genotyping quality score) less than 30 or not present; 4. HighDPFRatio to remove fraction of basecalls at a site greater than 0.4; 5. HighSNVSB to filter out SNVs with strand bias value (SB) exceeds 10; 6, 7. HighDepth and LowDepth to remove locus with depth greater than 3X the mean chromosome depth and locus depth below 3; 8. PloidyConflict to filter out genotype call from variant caller not consistent with chromosome ploidy. For the sorted tdTomato negative cells with CBE editing, variants that were also present in the parental H1-SOX2-H2B-tdTomato cells were filtered out to retain only de novo variants that generated by the editing tool.

For manuscripts utilizing custom algorithms or software that are central to the research but not yet described in published literature, software must be made available to editors and reviewers. We strongly encourage code deposition in a community repository (e.g. GitHub). See the Nature Portfolio guidelines for submitting code & software for further information.

Data

Policy information about availability of data

All manuscripts must include a data availability statement. This statement should provide the following information, where applicable:

- Accession codes, unique identifiers, or web links for publicly available datasets
- A description of any restrictions on data availability
- For clinical datasets or third party data, please ensure that the statement adheres to our policy

Human reference genome (GRCh38) was download from GenBank. Amplicon sequencing and whole-genome sequencing data are deposited in the NCBI BioProject ID: PRJNA812517. Source data is provided with this paper.

Human research participants

Policy information about studies involving human research participants and Sex and Gender in Research.

Reporting on sex and gender	This study are not related to human research participants.
Population characteristics	NA
Recruitment	NA
Ethics oversight	NA

Note that full information on the approval of the study protocol must also be provided in the manuscript.

Field-specific reporting

Please select the one below that is the best fit for your research. If you are not sure, read the appropriate sections before making your selection.

☒ Life sciences ☐ Behavioural & social sciences ☐ Ecological, evolutionary & environmental sciences

For a reference copy of the document with all sections, see nature.com/documents/nr-reporting-summary-flat.pdf

Life sciences study design

All studies must disclose on these points even when the disclosure is negative.

Sample size	No sample size calculation were performed. Sample size was determined to be adequate based on the consistency of measurable difference, and previously published literature in genome editing field, such as (Jonathan Strecker et al ,2019) and (Xiaoshu Xu et al, 2021).
Data exclusions	No data were excluded from the analyses.
Replication	RNA-seq and whole genome sequencing for CBE, with/without p53DD edited and sorted cells were performed in one set of biological samples (supple-fig. 5). All other experiments were performed at least two biological replicates. All the attempts at replication were successful.
Randomization	No randomization was performed. Cell culture conditions from all independent biological replicates were treated identical.
Blinding	Generally no blinding was performed as data are not subjective. Specifically, Miseq,Sanger sequencing,RNA-seq, and WGS were performed by 3rd party unaware of the treatment conditions. The gating of Flow cytometry analysis was set up based on negative control, and those gating conditions were applied to all samples.

Reporting for specific materials, systems and methods

We require information from authors about some types of materials, experimental systems and methods used in many studies. Here, indicate whether each material, system or method listed is relevant to your study. If you are not sure if a list item applies to your research, read the appropriate section before selecting a response.

Materials & experimental systems

n/a	Involved in the study
x	Antibodies
	x Eukaryotic cell lines
x	Palaeontology and archaeology
x	Animals and other organisms
x	Clinical data
x	Dual use research of concern

Methods

n/a	Involved in the study
x	ChIP-seq
	x Flow cytometry
x	MRI-based neuroimaging

Eukaryotic cell lines

Policy information about cell lines and Sex and Gender in Research

Cell line source(s)	H1 hESCs were purchased from WiCell Institute. The H1 SOX2-P2A-H2B-tdTomato reporter line were generated by knocking in a P2A-H2B-tdTomato transgene before the stop codon at the SOX2 gene locus using CRISPR/Cas9 based HDR using our previously reported method. The MSK-SRF001-iPSCs were generated from urine cells of an apparently healthy donor using our previously reported method. The 972-iPSCs were generated from a HGPS patient's fibroblast line purchased from Coriell Institute (AG01972), and the 756-iPSCs were generated from a PD patient's fibroblast line purchased from Coriell Institute (ND29756).
Authentication	H1 hESCs, MSK-SRF001-iPSCs, 972-iPSCs, 756-iPSCs, and the H1 SOX2-P2A-H2B-tdTomato reporter line were characterized with >99% pluripotent markers (OCT4/SOX2/NANOG) expression.
Mycoplasma contamination	All cell line was negative for mycoplasma contamination.
Commonly misidentified lines (See ICLAC register)	All the hESCs and hiPSCs have been fully characterized, and are not the commonly misidentified lines.

Flow Cytometry

Plots

Confirm that:

- [x] The axis labels state the marker and fluorochrome used (e.g. CD4-FITC).
- [x] The axis scales are clearly visible. Include numbers along axes only for bottom left plot of group (a 'group' is an analysis of identical markers).
- [x] All plots are contour plots with outliers or pseudocolor plots.
- [x] A numerical value for number of cells or percentage (with statistics) is provided.

Methodology

Sample preparation	Prior to FACS analysis, cells were detached using Accutase (Innovative Cell Tech. AT104) at 37°C for 10 min, followed by an addition of Stemflex Medium and centrifugation at 120 g for 3 min. Cell pellets were resuspended in 300 µl Stemflex, filtered through a Falcon 5 mL Round Bottom Polystyrene Test Tube with Cell Strainer Snap Cap (Fisher Scientific 352235), and kept on ice.
Instrument	BD FACSAria III
Software	BD FACSDiva Version 8.0.
Cell population abundance	The Supple-fig 5 is related to cell sorting. After creating a stop codon in the H2B reading frame by CBE with or without p53DD in the H1-H2B-tdTomato reporter cells, we isolated the edited cell population (tdTomato negative) by FACS sorting. The sorted cell were placed into cell culture and confirmed the silencing of tdTomato expression, under checking with Fluorescence microscope.
Gating strategy	Live cells were selected (side scatter area (SSC-A) versus forward scatter area (FSC-A)), followed by selection of single cells (side scatter width (SSC-W) versus side scatter height (SSC-H) then forward scatter width (FSC-W) versus height (FSC-H)). Either"GTG-GFP" vector electroporated cells or non-treated hESC lines were used as gating controls. The gating strategy is exemplified in Supplementary Fig. 18.

- [x] Tick this box to confirm that a figure exemplifying the gating strategy is provided in the Supplementary Information.

March 2021
